# Causal reasoning identifies mechanisms of sensitivity for a novel AKT kinase inhibitor, GSK690693

**DOI:** 10.1186/1471-2164-11-419

**Published:** 2010-07-06

**Authors:** Rakesh Kumar, Stephen J Blakemore, Catherine E Ellis, Emanuel F Petricoin, Dexter Pratt, Michael Macoritto, Andrea L Matthews, Joseph J Loureiro, Keith Elliston

**Affiliations:** 1 Oncology Biology, GlaxoSmithKline, 1250 South Collegeville Road, Collegeville, PA 19426, USA; 2 Center for Applied Proteomics and Molecular Medicine, George Mason University, Manassas, VA 20110, USA; 3 Genstruct, Inc., One Alewife Center, Cambridge, MA 02140, USA

## Abstract

**Background:**

Inappropriate activation of AKT signaling is a relatively common occurrence in human tumors, and can be caused by activation of components of, or by loss or decreased activity of inhibitors of, this signaling pathway. A novel, pan AKT kinase inhibitor, GSK690693, was developed in order to interfere with the inappropriate AKT signaling seen in these human malignancies. Causal network modeling is a systematic computational analysis that identifies upstream changes in gene regulation that can serve as explanations for observed changes in gene expression. In this study, causal network modeling is employed to elucidate mechanisms of action of GSK690693 that contribute to its observed biological effects. The mechanism of action of GSK690693 was evaluated in multiple human tumor cell lines from different tissues in 2-D cultures and xenografts using RNA expression and phosphoproteomics data. Understanding the molecular mechanism of action of novel targeted agents can enhance our understanding of various biological processes regulated by the intended target and facilitate their clinical development.

**Results:**

Causal network modeling on transcriptomic and proteomic data identified molecular networks that are comprised of activated or inhibited mechanisms that could explain observed changes in the sensitive cell lines treated with GSK690693. Four networks common to all cell lines and xenografts tested were identified linking GSK690693 inhibition of AKT kinase activity to decreased proliferation. These networks included increased RB1 activity, decreased MYC activity, decreased TFRC activity, and increased FOXO1/FOXO3 activity.

**Conclusion:**

AKT is involved in regulating both cell proliferation and apoptotic pathways; however, the primary effect with GSK690693 appears to be anti-proliferative in the cell lines and xenografts evaluated. Furthermore, these results indicate that anti-proliferative responses to GSK690693 in either 2-D culture or xenograft models may share common mechanisms within and across sensitive cell lines.

## Background

Hyperactivation of the PI3K-AKT pathway is one of the most common molecular findings in human malignancies [1,2]. Constitutive activation of this pathway can result from several factors, including mutation and/or amplification in certain components within this pathway, e.g., EGFR, ERBB2, PI3K, and AKT as well as the downregulation or loss of negative regulators such as the serine phosphatase, PTEN [3,4]. Increased AKT1 activity has been observed in approximately 40% of breast and ovarian cancers and >50% of prostate carcinomas. Activation of AKT2 kinase has been observed in 30-40% of ovarian and pancreatic cancers [3,5]. Increased AKT3 enzymatic activity was found in estrogen receptor-deficient breast cancer and androgen insensitive prostate cancer cell lines, suggesting that AKT3 may contribute to the aggressiveness of steroid hormone-insensitive cancers [3]. AKT signaling has been reported to promote cell survival and proliferation across different cell types and can involve multiple downstream mechanisms including activation of FRAP1 (mTOR)/P70S6K1, inactivation of CDKN1B (p27Kip), inactivation of Forkhead family transcription factors, and increased cyclin D1 (CCND1). In breast cancer cells, the anti-proliferative function of the PTEN tumor suppressor protein has been demonstrated to involve the inhibition of AKT-mediated cell cycle activation through both its protein and more canonical lipid phosphatase activities and the function of the CDKN1B cell cycle inhibitor has been shown to be directly inhibited by AKT-dependent phosphorylation [6-9]. In ovarian cancer cells, PI3K/AKT signaling has been demonstrated to affect cell proliferation via FRAP1(mTOR)/P70S6K1-mediated mechanisms [10,11]. Proliferation of embryonic cardiomyocytes in cell culture has been demonstrated to be dependent on PI3K/AKT signaling leading to inhibition of the activity of the Forkhead family transcription factors, FOXO1A and FOXO3A [12]. In rat and mouse cell lines, MYC-induced proliferation and transformation was shown to require AKT-mediated phosphorylation and inhibition of Forkhead family proteins.

AKT provides survival signals through inhibiting several proapoptotic factors in the caspase cascade, including BAD, (pro)caspase-9, PEA15 (PED), CDKN1A (p21/WAF1), and MAP3K5 (ASK1) [3]. AKT also regulates apoptosis by providing positive and negative transcriptional signaling. Phosphorylation of FOXO by AKT prevents its nuclear entry and thereby preventing transcription of proapoptotic genes, including Fas ligand, BIM, TRAIL and TRADD. In contrast [13], AKT promotes nuclear translocation of NF-κB by phosphorylating and activating IκB kinase (IKK), leading to the phosphorylation and proteosomal degradation of IκB (inhibitor of NF-κB), and ultimately NF-κB nuclear localization. AKT can also inactivate p53 by modulating subcellular localization of Mdm2. Phosphorylation of Mdm2 by AKT is necessary for localization to the nucleus, where Mdm2 can complex with p53 to promote its ubiquitin/proteasome-mediated degradation [14].

The present study investigated mechanisms induced in cancer cell lines in response to treatment with GSK690693, an ATP-competitive, pan-AKT kinase inhibitor with potent enzyme and cellular activity being investigated in patients with solid tumor and hematological malignancies [15]. The goal of this study was to identify mechanisms that were common to a set of cell lines sensitive to GSK690693 and to evaluate those mechanisms for their potential effects on cell survival and proliferation. Candidate mechanisms were identified via analysis of gene expression profiling data and phosphoproteomic data using a causal network modeling methodology called "Reverse Causal Analysis" (RCA) [16,17]. RCA enables the mechanistic interpretation of large datasets using a large network model of biological cause and effect relationships. In RCA, the causal network upstream from entities observed to change in the experiment (such as RNA abundances measured by microarray) is automatically evaluated to identify and rank many thousands of subnetworks that express potential mechanistic explanations for the observed changes. Biologists using RCA methodology can then construct a Causal Network Model (CNM) where multiple mechanism networks and their supporting evidence are merged to form a unified causal network consistent with the input datasets. The CNM represents the set of hypotheses explaining the observed changes. The CNM in the present study is further constrained to contain only those subnetworks that are supported by the observed changes in all sensitive cell lines: it represents the common set of hypotheses for mechanisms of sensitivity to GSK690693.

## Results

### Inhibition of cellular AKT activity by GSK690693

**Figure 1:**
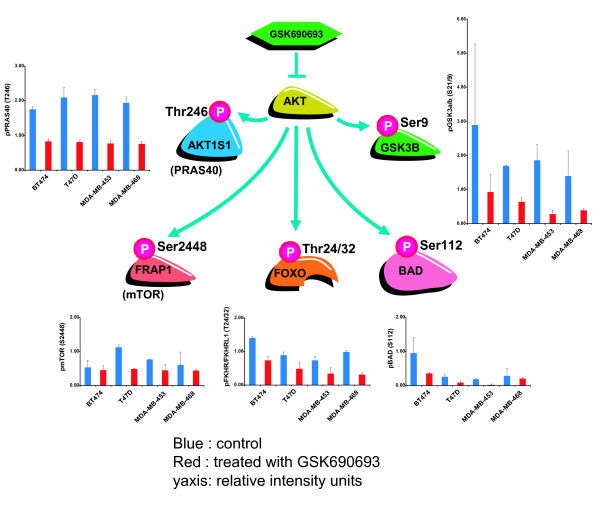
**Treatment with GSK690693 leads to inhibited phosphorylation of kinase targets of AKT**. Inhibition of AKT kinase activity is evidenced by decreased phosphorylation of kinase targets of the protein, including PRAS40 (AKT1S1), mTOR (FRAP1), FKHR/FKHRL1 (FOXO1A/FOXO3A), BAD and GSK3a/b in BT474, MDA-MB-453, MDA-MB-468, and T47D breast cancer cell lines, mean +/- S.D. *Bar graphs: blue: control, red: treated with GSK690693; y-axis: relative intensity units*.

GSK690693 inhibits proliferation of certain tumor cell lines *in vitro *and attenuates the growth of human tumor xenografts in mice [15]. To investigate the molecular cascade leading to the inhibition of cell proliferation by GSK690693, various tumor cell lines growing in cell culture or as xenografts in mice were treated with vehicle or GSK690693. Cell lines used in this study were either classified as sensitive to GSK690693 (IC50 < 200 nM): T47D, BT474, and LNCaP or less sensitive (IC50 > 800 nM): MDA-MB-453, MDA-MB-468, and SKOV3 [15]. Daily treatment of mice with GSK690693 inhibited growth of BT474, LNCaP, and SKOV3 tumor xenografts [15]. Microarray analysis was used to generate RNA profiles for the cell culture experiments (BT474, T47D, MDA-MB-468, MDA-MB-453, LNCaP, and SKOV-3) as well as for the xenograft experiments (BT474, LNCaP, and SKOV-3). Changes in the phosphorylation of various proteins following GSK690693 treatment were analyzed for the BT474, T47D, MDA-MB-468, and MDA-MB-453 breast cancer cells in cell culture using reverse phase protein array (RPPA). Treatment with GSK690693 resulted in a decrease in the abundance of phosphorylated AKT substrates, pGSK3a/b (S9/21), pFKHR/FKHRL1 (T24/32), pmTOR (S2448), pBAD (S112), and pPRAS40 (T246) in BT474, T47D, MDA-MB-468, and MDA-MB-453 cells (Figure 1), providing direct evidence for the inhibition of AKT kinase activity by GSK690693. Causal network modeling was used to identify chains of causation linking the upstream perturbation to downstream hypotheses identified by RCA incorporating phosphoproteomic and transcriptomic changes as evidence. For example the observed decrease in GSK3B and FRAP1 phosphorylations support decreased MYC activity in response to AKT inhibition.

### AKT inhibition can increase transcriptional activity of FOXO1A and FOXO3A

**Figure 2:**
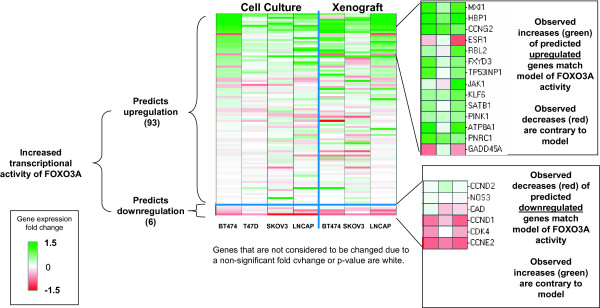
**Changes in RNA expression levels of genes controlled by FOXO3A support transcriptional activation of FOXO**. *Upper Panels: *Genes that are consistent with increased transcriptional activity FOXO3A are green, those inconsistent are red. *Lower panels: *Genes that are consistent with increased transcriptional activity FOXO3A are red, those inconsistent are green. *Both panels: *Genes that are not considered to be changed due to a non-significant fold change or p-value are white. Genes which are inconsistent with the predicted activity are represented in Additional file 1 Table S1 with an "X".

AKT directly phosphorylates FOXO1A and FOXO3A at T24/S256 and T32/S253, respectively, excluding the proteins from the nucleus and effectively inhibiting their transcriptional activities [18,19]. Thus, inhibition of AKT by GSK690693 can increase FOXO transcriptional activity, by decreasing its phosphorylation. Decreased phosphorylation of FOXO1A/FOXO3A was observed in the BT474, T47D, MDA-MB-453, and MDA-MB-468 cell culture experiments after treatment with GSK6906936 (Figure 1). RCA of RNA expression changes supports increased transcriptional activity of FOXO3A in all three xenografts and FOXO1A in two of the three xenografts (BT474 and LNCaP). RCA of RNA expression changes supports the increased transcriptional activity of both FOXO1A and FOXO3A in the BT474, T47D, SKOV-3, and LNCaP cells treated in culture (Figure 2). There is approximately a 50% overlap between the RNA expression changes explained by the two *FOXO *family members, and collectively they explain 31, 35, and 45 RNA expression changes observed after three days of drug treatment in BT474, SKOV-3, and LNCaP xenografts, respectively (Additional file 1 Table S1 contains a summary table of RNA expression changes in these studies). Increased transcriptional activity of FOXO1A and FOXO3A also explains 87, 59, 89, and 107 transcriptional changes in BT474, T47D, SKOV-3, and LNCaP cultured cells, respectively (BT474 and T47D cells 24/48 hours pooled, SKOV-3 and LNCaP cells at 24 hours) (Figure 2, Additional file 2 Table S2). Increased transcriptional activity of FOXO1A and FOXO3A is not supported by RCA in the two cell lines less sensitive to GSK690693 MDA-MB-453 and MDA-MB-468 cells (Data not shown). Increased nuclear translocation of a FOXO3--green florescent protein hybrid in response to GSK690693 treatment in U2OS cells has been shown previously, in confirmation of these results [15].

### Decreased MYC transcriptional activity

**Figure 3:**
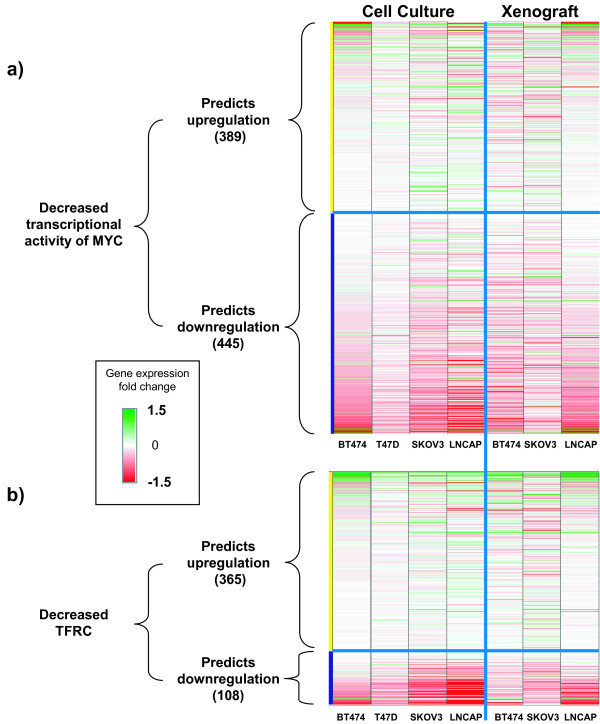
**Inhibition of MYC and consequent inhibition of TFRC**. **A) ***Upper Panels: *Genes that are consistent with decreased transcriptional activity MYC are green, those inconsistent are red. *Lower panels: *Genes that are consistent with decreased transcriptional activity MYC are red, those inconsistent are green. **B) ***Upper Panels: *Genes that are consistent with decreased TFRC activity are green, those inconsistent are red. *Lower panels: *Genes that are consistent with decreased TFRC activity are red, those inconsistent are green. *All panels: *Genes that are not considered to be changed due to a non-significant fold change or p-value are white. Genes that are inconsistent with the predicted activity are represented in Additional file 2 Table S2 and Additional file 8 Table S5 with an "X".

RCA identifies RNA expression changes that strongly support decreased transcriptional activity of MYC in all three xenografts as well as in four sensitive tumor cell lines in culture. Inhibition of AKT can lead to decreased transcriptional activity of MYC through multiple mechanisms. Decreased MYC activity can correctly explain 31, 61, and 54 RNA expression changes in the BT474, SKOV-3, and LNCaP xenografts, respectively (Figure 3, Additional file 3 Table S3). Decreased MYC activity is also supported by RNA expression changes in BT474 and T47D (24/48 hours pooled),,SKOV-3, and LNCaP cultured cells at 24 hours (Figure 3, Additional file 3 Table S3). Decreased transcriptional activity of MYC is not supported by RCA in the MDA-MB-453 and MDA-MB-468 cells (Data not shown).

Although MYC RNA levels are not observed to be significantly changed in any cell line at any time point following drug treatment (Additional file 4 Figure S1), multiple mechanisms can link AKT inhibition to decreased MYC protein abundance and transcriptional activity. In the SKOV-3 cell culture experiment, evidence supporting decreased MYC activity is observed as early as two hours after treatment (Additional file 5 Table S4) and in the LNCaP cell culture experiment at eight hours after treatment, consistent with fast, post-translational control of MYC. AKT directly phosphorylates GSK3β leading to inhibition of its kinase activity, and decreased GSK3β phosphorylation is observed in response to AKT inhibition by GSK690693 treatment (Figure 1). Active GSK3β phosphorylates MYC at residue T58, thereby targeting MYC for degradation by FBXW7 [20]. AKT phosphorylates FRAP1 (mTOR) at S2448 increasing FRAP1 kinase activity, and decreased FRAP1 S2448 phosphorylation was observed in the breast cancer cell lines (Figure 1) [21]. Additionally, AKT directly phosphorylates AKT1S1 (PRAS40), and this phosphorylation acts to block the inhibitory binding of AKT1S1 to FRAP1 [22]. Decreased phosphorylation of AKT1S1 at T246 was observed in all four breast cancer cell lines in response to GSK690693 treatment (Figure 1). Increased FRAP1 activity can lead to increased activity of EIF4E, a translation initiation factor that directly increases MYC translation, decreasing FRAP1 activity that could result in decreased protein abundance of MYC [23].

FOXO3A/FOXO1A transcriptional activities can inhibit the induction of multiple MYC target genes [24] and can upregulate the expression of specific cell cycle-inhibitory genes that are also downregulated by MYC activity, such as *HBP1*, *CCNG2*, and *CDKN1B*. RNA expression of these three genes was increased in multiple cell culture and xenograft experiments, consistent with increased FOXO1A/FOXO3A and decreased MYC transcriptional activity (Additional file 6 Figure S2).

Proteins of the MAD family are another potential regulator of MYC activity [25]. Transcript levels of *MXI1*, a MAD family member that inhibits MYC activity, were observed to be increased in response to GSK690693 treatment in SKOV-3, BT474, and LNCAP cell culture experiments and in BT474 xenografts (Additional file 7 Figure S3). This is consistent with recent findings in DLD-1 colon carcinoma cells in which AKT2 silencing resulted in increased FOXO3A activity leading to increased *MXI1 *RNA expression [26].

### Decreased TFRC activity

RCA identified RNA expression changes that support decreased TFRC activity in SKOV-3 and LNCaP xenografts and in T47D, SKOV-3, and LNCaP cultured cells (Figure 3, Additional file 8 Table S5). Increased AKT activity can increase TFRC cell surface expression, leading to increased TFRC activation [27]. TFRC has recently been reported to be a direct transcriptional target of MYC, and TFRC was shown to be required for MYC-mediated cell proliferation [28], providing a direct mechanism by which AKT can mediate TFRC cell surface expression. TFRC RNA levels were decreased in response to treatment in the SKOV-3 xenograft, and in BT474, SKOV-3, and LNCaP cell culture experiments (Additional file 9 Figure S4). Decreased TFRC activity explains 43 and 34 RNA expression changes in the SKOV-3 and LNCaP three day xenografts, respectively. Decreased TFRC activity also explains 66, 90, and 125 RNA expression changes in T47D, SKOV-3, and LNCaP cultured cells (Figure 3, Additional file 8 Table S5). Decreased TFRC activity is not supported by RCA in the MDA-MB-453 and MDA-MB-468 cells (Data not shown).

### RB1-mediated G1 cell cycle arrest

**Figure 4:**
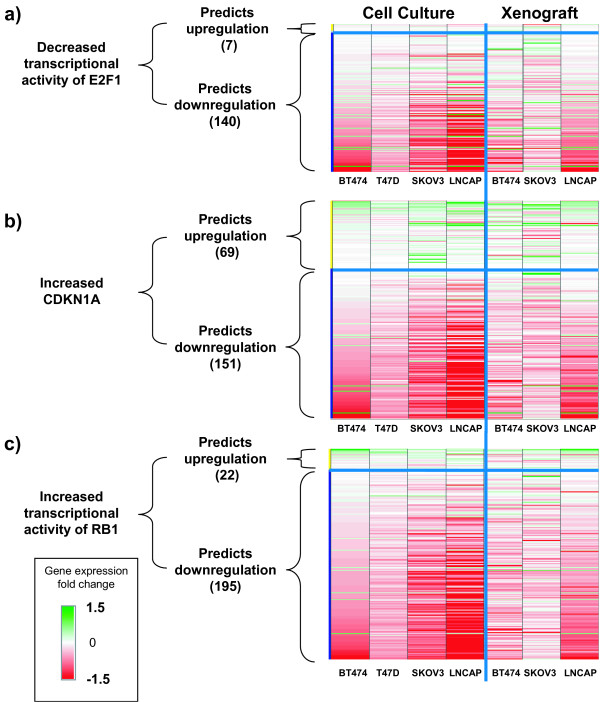
**Regulation of cell cycle controllers is evidenced by changes in RNA expression levels**. **A) ***Upper Panels: *Genes that are consistent with decreased transcriptional activity of E2f1are green, those inconsistent are red. *Lower panels: *Genes that are consistent with decreased transcriptional activity of E2f1 are red, those inconsistent are green. **B) ***Upper Panels: *Genes that are consistent with increased activity of CDKN1A are green, those inconsistent are red. *Lower panels: *Genes that are consistent with increased activity of CDKN1A are red, those inconsistent are green. **C) ***Upper Panels: *Genes that are consistent with increased transcriptional activity of RB1 are green, those inconsistent are red. *Lower panels: *Genes that are consistent with increased transcriptional activity of RB1 are red, those inconsistent are green. *All panels: *Genes that are not considered to be changed due to a non-significant fold change or p-value are white. Genes that are inconsistent with the predicted activity are represented in Additional files 10, 11 and 12 Tables S6-S8 with an "X".

RCA identifies RNA expression changes that support increased cell cycle arrest in all three xenografts and RNA expression and phosphoprotein changes that support increased cell cycle arrest in BT474, T47D, SKOV-3, and LNCaP cultured cells (Figure 4). RNA expression changes strongly support an increase in cell cycle arrest based on evidence for increased activity of cell cycle suppressors CDKN1A, RB1, and E2F4, and decreased activity of cell cycle activators E2F1, E2F2, and E2F3 (Figure 4).

RB1 is a cell cycle regulatory protein that can inhibit the G1/S transition [29] and the M/G1 transition [30]. The activation of RB1-mediated transcription and its effects on this regulatory network by GSK690693 can collectively explain 13, 36, and 33 RNA expression changes in the BT474, SKOV-3, and LNCaP xenografts, respectively, at three days. The activation of RB1 also explains 81, 103, 139, and 154 RNA expression changes in the BT474, T47D, SKOV-3, and LNCaP cell cultures (Figure 4, Additional file 10 Table S6). The activation of RB1 is not supported in the MDA-MB-453 and MDA-MB-468 cells (Data not shown).

AKT inhibition can lead to RB1 activation and cell cycle arrest through multiple independent mechanisms including activation of the cell cycle inhibitor CDKN1A [31-33]. AKT can directly phosphorylate CDKN1A inhibiting the activity of this protein by secluding it in the cytoplasm [34]. Increased CDKN1A activity due to AKT inhibition is strongly supported by 16, 36, and 30 RNA expression changes in the BT474, SKOV-3, and LNCaP xenograft three day experiments, respectively. Increased CDKN1A activity also explains 58, 84, 114, and 121 RNA expression changes in the BT474, T47D, SKOV-3, and LNCaP cell culture experiments, but is not supported in the MDA-MB-453 and MDA-MB-468 cell culture experiments (Figure 4, Additional file 11 Table S7, Data not shown).

The E2F family of transcription factors regulates cell cycle progression, with the activities of E2F1, E2F2, and E2F3 promoting cell cycle progression and the activity of E2F4 inhibiting cell cycle progression [35]. Decreased E2F1 transcriptional activity is supported by RNA expression changes in all three xenograft experiments (10, 17, and 11 RNA expression changes in BT474, SKOV-3, and LNCaP, respectively) as well as in cultured cells (49, 51, 70, and 76 in BT474, T47D, SKOV-3, and LNCaP, respectively) (Figure 4, Additional file 12 Table S8). Decreased transcriptional activity of E2F2 and E2F3, and increased transcriptional activity of E2F4 are supported by RNA expression changes in all three xenografts and BT474, T47D, SKOV-3, and LNCaP cells (Data not shown). Increased transcriptional activity of E2F4 and decreased transcriptional activities of E2F1, E2F2, and E2F3 are not supported by RCA in the MDA-MB-453 and MDA-MB-468 cells (Data not shown).

### Distinct sets of gene expression changes support the same hypotheses, and the hypotheses form a Causal Network Model providing a mechanism that links AKT inhibition with reduced proliferation

**Figure 5:**
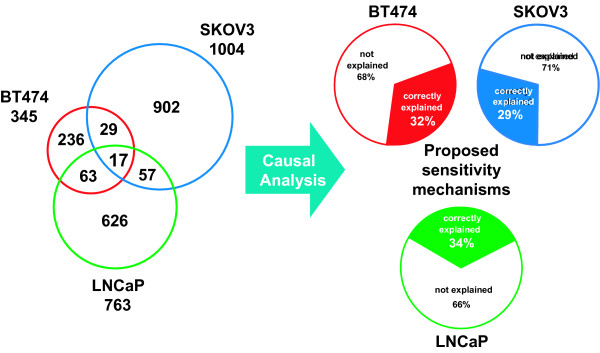
**Different gene changes support the same processes in cell culture and xenograft experiments**. The transcriptomic response to GSK690693 treatment is markedly different in three xenograft models derived from distinct tissues, with only 17 common expression changes (<1% of total). In contrast, a common set of proposed sensitivity mechanisms can explain roughly one-third of the expression changes in each cell line.

**Figure 6:**
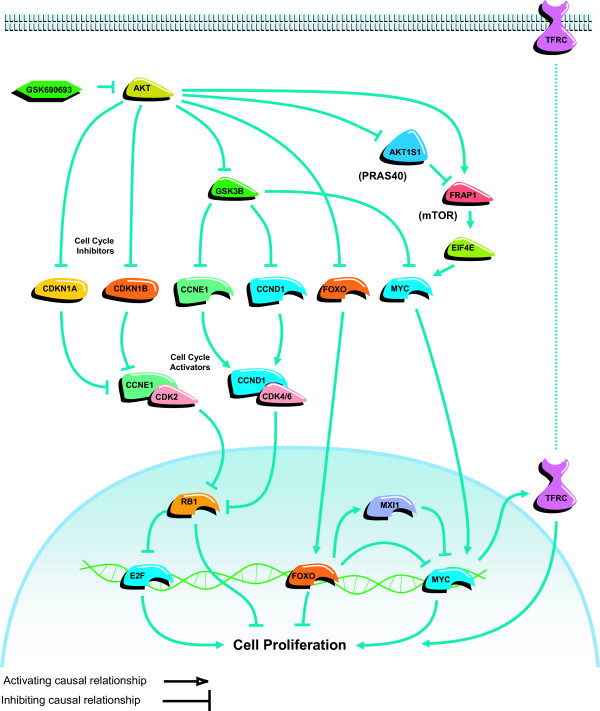
**Summary of processes which are affected by GSK690693 treatment**. Activating causal relationship; Inhibiting causal relationship. Note that this is a summary representation: in the actual Causal Network Model protein activities are differentiated from protein abundances and protein modifiers such as phosphorylations.

As represented in Figure 5, three very distinct sets of RNA expression changes were identified in response to GSK690693 treatment in three xenograft experiments. Seventeen RNA expression changes were common to all three xenograft experiments. However, the hypotheses of increased FOXO activity, increased cell cycle arrest, decreased MYC transcriptional activity, and decreased TFRC activity correctly explain 31%, 26%, and 31% of the RNA expression changes in each of the three xenograft experiments (BT474, SKOV-3, and LNCaP), respectively. Similar explanatory power of these hypotheses is seen in the cell culture experiments, in which 33%, 50%, 45%, and 32% of RNA expression changes were correctly explained in BT474, T47D, SKOV3, and LNCaP cells, respectively. In contrast to the small overlap in RNA expression changes, the four mechanisms supporting GSK690693 driven anti-proliferative effects identified by RCA form a Causal Network Model that can correctly explain a large portion of the RNA expression changes identified in each xenograft and cell culture experiment (Figure 6). The power of causal analysis is exemplified by the expression changes related to two cell cycle marker genes, PCNA and MKI67. Although the increased cell cycle arrest hypothesis was well supported in all experiments, changes in these two genes failed to meet significance criteria in one or more of the xenograft experiments. Each of the four mechanisms is well supported by RNA expression changes from each xenograft and cell culture experiment; however the specific expression patterns supporting the mechanisms are minimally overlapping.

## Discussion

The AKT family of kinases has been well characterized as a mediator of cell proliferation and survival, and these functionalities highlight the role of these kinases in tumor progression [2,3]. In this study, we characterized molecular signaling networks that are activated or inhibited by AKT inhibition by GSK690693 treatment using gene microarray data from multiple cell lines and xenograft models and phosphoproteomics analysis from multiple cell lines.

The focus of this analysis was to identify common mechanisms of action for GSK690693 treatment in breast, prostate, and ovarian cancer cell lines in both cell culture and xenograft models. RCA enabled the analysis of large, transcriptomic data sets in combination with a more targeted phosphoproteomics data set to identify mechanisms of action for this AKT inhibitor that were supported by specific RNA expression and phosphoprotein changes following GSK690693 treatment.

Treatment with GSK690693 inhibited the kinase activity of AKT, and led to alterations in multiple downstream signaling pathways (Figure 1). The four hypotheses identified by RCA form a Causal Network Model in which inhibition of AKT leads to cell cycle arrest and inhibition of proliferation (Figure 6). The increased transcriptional activity of FOXO1A and FOXO3A can directly increase the expression of cell cycle inhibitors, such as HBP1, CCNG2, and CDKN1B, and these genes were upregulated in the majority of the sensitive cell culture and xenograft experiments (Figure 2). The expression of these three genes is repressed through the activity of MYC. The pro-proliferative effect of MYC activation is well established [36], and repression of the transcriptional activity of this protein, which was supported by RCA, would lead to decreased cell proliferation (Figure 3). TFRC is a MYC-regulated gene that was observed to be decreased in response to GSK690693 treatment in some of the cell lines and xenografts. Furthermore, decreased protein abundance of TFRC was supported by RCA, and TFRC cell surface expression has been shown to be greater in cancer cells than in normal cells [37] and a positive correlation has been reported between the number of cell surface transferrin receptors and the rate of cell proliferation [38-40]. Inhibition of TFRC decreases cell proliferation and results in G1 arrest, consistent with the tumor growth inhibition observed in the sensitive cell culture and xenograft experiments (Figure 3). AKT inhibition can also directly stimulate cell cycle arrest through attenuation of the direct inhibitory AKT-mediated phosphorylation of the cell cycle inhibitors CDKN1A and CDKN1B, as well as through modulation of the activity of the signaling intermediate GSK3β (Figure 4). In combination, these four hypotheses describe a mechanism for inhibition of proliferation due to AKT inhibition, primarily through cell cycle arrest and identified inhibition of proliferation, as opposed to the more canonical survival role attributed to AKT signaling, as the primary process responsible (Figure 6).

AKT activation results in both anti-apoptotic and pro-proliferative signals, although the evidence for induction of apoptosis was generally lacking or weak in our study with the AKT kinase inhibitor, suggesting that AKT plays a more critical role in regulating cell proliferation in epithelial cancer cells. This is in contrast to findings in lymphoblastic leukemia cells lines treated with GSK690693 in which caspase 3/7 induction and concomitant increased sub-2N populations were observed in addition to decreased proliferation [41]. Treatment with GSK690693 resulted in increased FOXO1A and FOXO3A transcriptional activity, although the transcript levels of pro-apoptotic genes (e.g., BCL2L11 and TNFSF10) were not consistently upregulated in most of the cell culture and xenograft models. Similarly, causal analysis did not identify change in the NF-κB transcriptional activity in any of the model systems upon treatment with GSK690693. Further, phosphorylation of BAD was decreased in cells treated with GSK690693 (Figure 1), suggesting regulation of apoptotic pathways. Although there was some evidence of apoptosis in LNCaP and BT474 cells at 24-48 h (data not shown), the other cell lines did not show evidence of this process. Taken together, our data suggest that inhibition of AKT kinases can regulate both cell proliferation and apoptotic pathways. This is consistent with previous findings that GSK690693 treatment inhibited tumor formation in a mouse model that spontaneously develops lymphomas through both induction of apoptosis as well as inhibition of proliferation [42]. However, the primary mechanism of action of GSK690693 observed in this study appears to be anti-proliferative in the cell lines and xenografts evaluated. Collectively these data indicate that the balance of the role of AKT signaling in either cell survival or proliferation is likely to be dependent on tumor type.

In this study, independent identification of hypotheses supported by a preponderance of evidence in each cell culture and xenograft experimental model enabled the comparison of the drug mechanism of action in each distinct experimental model at the hypothesis level. Further, as evidenced in this study, while treatment with the same compound can lead to large and mostly non-overlapping changes in cell lines and xenografts from various histological origins, underlying mechanisms of action are conserved (Figure 5). Interestingly, as demonstrated in Figure 5, the Causal Network Model of common mechanisms of sensitivity to GSK690693 derived by RCA only accounted for between 29-34% of the RNA-based xenograft changes (Figure 5) (similar coverage was also achieved by RCA on 2-D culture data (not shown)). This suggests that there are clearly additional mechanisms of response to GSK690693 that are not accounted for by the current causal model. There are several factors that may account for this observation. First, the goal of the analysis was to identify conserved mechanisms of response in sensitive cell lines, thus excluding those mechanisms supported by the data that were unique to specific cell lines. Additionally, as the RCA methodology is based on the causal knowledge modeled for each gene expression change that is linked to underlying literature knowledge of each transcript/protein, therefore expression changes where such information is sparse are less likely to contribute significantly to the causal model.

## Conclusions

We have demonstrated the mechanism of action of a novel AKT kinase inhibitor, GSK690693, using a Causal Network Model, that allows a diverse data set to be explained by common hypotheses. Inhibition of AKT kinases in cell culture and tumor xenografts results in cell cycle inhibition by altering various cellular mechanisms, which are interrelated. These mechanisms include increasing FOXO transcriptional activity, inhibition of MYC transcriptional activity, decreased TFRC activity, and induction of RB1-mediated cell cycle arrest. Our findings demonstrate one of the main tenets of systems biology, that the networks that regulate cellular processes are often conserved across various tumor and tissue types, even when the transcriptional response of these tissues is markedly different.

## Methods

### GSK690693 preparation

GSK690693 was synthesized at GlaxoSmithKline. For all *in vitro *studies, GSK690693 was dissolved in DMSO at a concentration of 10 mM prior to use and subsequently diluted in aqueous medium. For the tumor xenograft studies, GSK690693 was formulated in either 4% DMSO/40% hydroxypropyl-β-cyclodextrin in water, pH6.0.

### Animals

Female CD1 Swiss Nude mice were obtained from Taconic (Hudson, NY) and C.B-17 SCID mice were obtained from Charles River (Wilmington, MA). All animal studies were performed in compliance with federal requirements, GlaxoSmithKline policy on the Care and Use of Animals, and with related codes of practice.

### Sample preparation for RNA and Phosphoprotein analysis

Human tumor cell lines BT474, T47D, MDA-MB-468, MDA-MB-453, LNCaP, and SKOV-3 were treated with 1 μM GSK690693 for 2, 8, 24, and 48 h (N = 3 or 4 replicates/treatment group) and lysates were prepared in Trizol for RNA expression analysis. Phosphorylation of various AKT substrates was analyzed in breast carcinoma cell lines treated for 10 min, 30 min, 2 h, or 24 h of treatment (N = 2 replicate/time point) using reverse phase protein microarray.

Tumor xenografts were initiated by injection of tumor cell suspension (LNCaP) or tumor fragments (BT474, SKOV-3) subcutaneously in 8-12 week old CD1 Swiss Nude mice (LNCaP, SKOV-3) or SCID mice (BT474). When tumors reached a volume of 100-200 mm^3^, mice were randomized and divided into groups of 8-12 mice/group. GSK690693 was administered once daily at 30 mg/kg by IP administration. Tumor tissues were harvested after dosing for 3, 7, or 21 days (n = 3 mice/group/time point) and homogenized in Trizol. Total RNA was isolated from each Trizol lysate using RNeasy reagents (Qiagen, Valencia, CA) and was quantified by spectroscopy and quality assessed using an Agilent Bioanalyser. Five micrograms of high quality total RNA was used to generate amplified cRNA probe material using the Eberwine protocol (Van Gelder *et al.*, 1990) and hybridized overnight using U133 Plus 2.0 GeneChips (Affymetrix, Santa Clara, CA). GeneChip washing and scanning were performed according to manufacturer's instructions.

### Reverse-phase Protein Array

Protein arrays were constructed as described previously [15]. Briefly, serially diluted protein lysates were printed in duplicate onto nitrocellulose-coated glass slides. The lysate arrays were incubated for at least 5 hours in blocking solution [1 g I-block (Tropix, Bedford, MA), 0.1% Tween-20 in 500 mL PBS] at room temperature with constant rocking. Blocked arrays were stained with pGSK3a/b (S9), pFOXO (T24/32), pFOXO (S256), pmTOR (S2448), pBAD (S112), and pPRAS40 (T246) antibody on an automated slide stainer. All antibodies were obtained from Cell Signaling Technology (Beverly, MA), except phospho-PRAS40 antibody which was purchased from Biosource (Carlsbad, CA). Stained slides were scanned individually on a UMAX PowerLook III scanner (UMAX, Dallas, TX, USA) at 600 dpi and saved as TIF files in Photoshop 6.0 (Adobe, San Jose, CA, USA). The TIF images for antibody-stained slides and Sypro-stained slide images were analyzed with MicroVigene image analysis software, version 2.200 (Vigenetech, North Billerica, MA) and Microsoft Excel 2000 software. Images were imported into Microvigene, which performed spot finding, local background subtraction, replicate averaging, and total protein normalization, producing a single value for each sample at each endpoint.

### Data Analysis

Microarray data was analyzed using the R-based Bioconductor suite of analytical tools to determine genes that changed state across a variety of comparisons. RMA analysis was applied to generate expression values from the Affymetrix CEL files. Principle component analysis was applied to the expression values for each group of microarrays to determine if any samples differed dramatically from the set of similar microarrays. Significant gene expression changes for all but the LNCaP 3 day xenograft comparison were generated based on ANOVA adjusted p-values of 0.05 corrected for multiple testing effects using Benjamini-Hochberg FDR and fold changes of at least 1.3. As the purpose of this analysis was to determine common mechanisms of action across diverse cell lines and xenografts, the choice of a conservative minimum fold change selection of 1.3 was appropriate in that it ensured that hypotheses were supported by clear experimental evidence. Due to the large number of fold changes in the LNCaP 3 day xenografts an unadjusted p-value of 0.01 was used without a fold change criteria.

Phosphoproteomic values were determined using reverse phase proteomic analysis. The resulting values were analyzed using the ratios of the treated and vehicle cell lines at 10 and 30 minutes, 2 and 24 hours. The presence of a significant change was determined by a 20 percent decrease or a 50 percent increase in the treated compared to the vehicle samples. These cutoff thresholds were determined empirically to enable changes to contribute to the analysis across cell lines.

### Causal Reasoning Methodology

In this study, the activation or inhibition of specific biological signaling networks were identified as explanations for statistically significant RNA gene expression changes observed in response to treatment with GSK690693 in multiple cell lines. These networks represent mechanistic hypotheses for molecular effects of GSK690693 and together they comprise a network called a Causal Network Model (CNM) that links GSK690693 treatment to a large fraction of the observed data in multiple experiments via common mechanisms. These networks were identified in a two-stage process: (1) Reverse Causal Analysis, an automated analysis of the experimental data using a large, literature-derived network of cause-and-effect relationships, the Genstruct Human Knowledge Assembly Model, and (2) a software-assisted methodology enabling scientists to vet the results of the automated analysis and to produce the explanatory networks. Note that this process is a means to explain observed data in the context of existing knowledge, distinct from approaches that attempt to infer novel causal relationships from observed data.

The Human Knowledge Assembly Model (KAM) is a set of human-specific causal assertions that has been augmented with orthologous causal assertions derived from either rat or mouse sources. Each causal assertion is the result of manual curation of the scientific literature and is supported by one or more specific scientific citations. An example causal assertion would be: increased transcriptional activity of NF-KB complex causing an increase in the gene expression of the insulin receptor substrate 1 (Irs1) (Ruan et al., 2002).

Reverse Causal Analysis (RCA) of experimental data evaluates each node in the KAM as a hypothesis, a potential cause for observed differential measurements in an experiment. By computing statistical figures of merit for each hypothesis, RCA enables each hypothesis to be ranked by multiple criteria (see below) and prioritized for inclusion in larger explanatory networks. RCA starts with the quantification of differential measurements as "state changes", reducing values to be one of "increase", "decrease" or "no change". While these differences are referred to as "changes" , in fact they can be any differences in state between two biological systems, such as differential protein expression between drug-sensitive and insensitive cell lines, or differential RNA expression between tissues of knockout and wild-type animals. State changes are then assigned to nodes in the KAM that represent entities corresponding to the measurements. In the case of transcriptomic data, state changes are mapped to nodes representing RNA abundances. Finally, every node in the KAM is evaluated as a hypothesis, where a hypothesis is a potential explanation for some subset of the state changes. A node evaluated as a hypothesis is the "root" of the hypothesis and the hypothesis is composed by assuming that the root node has a value of "increased" and then searching a defined number of steps in the network of the KAM for all causal paths leading from the root node to a mapped state change. Each state change node found by this search is a "prediction" of the hypothesis and is assigned a polarity of either "increase", "decrease", or "ambiguous". The polarity assignment is based on the sequence of inverting and non-inverting causal relationships traversed in each path from the root node to the state change node. If the state change node can be reached by paths making contradictory polarity assignments, it is assigned a polarity of "ambiguous". The set of predictions for each hypothesis is then evaluated with respect to the mapped state changes by calculating two figures of merit: Richness and Concordance p-values. Richness is a measure of the relevance of a hypothesis to the changes observed in the experiment, while Concordance is a measure of the accuracy of the predictions of a hypothesis. Both Richness and Concordance cast the predictions and the measurement data into canonical forms for probability analysis. Richness is a measure of the over-representation of observed state changes in the set of genes for which a hypothesis makes predictions. For example, observed state changes are overrepresented if only 1% of all genes measured show significant change but 10% of the genes for which a hypothesis makes predictions show significant change. Richness is the significance of the overrepresentation, calculated as a p-value based on the hypergeometric distribution, i.e. sampling without replacement. It is the likelihood of having Q state changes both predicted to change and observed to change, given N total measurements, M total significant changes and P predictions. Note that Richness does not depend on whether the observed direction of any change agrees with the predicted direction; hence ambiguous predictions may be included in the calculation. Concordance is a measure of the correctness of the predictions of the hypothesis, whether observed changes agree with unambiguous predictions. The direction of the hypothesis root is taken to be the direction that results in the higher number of successful predictions. The stronger the biases of the supporting evidence in favor of a hypothesis root direction, the more concordant the hypothesis. The significance of this bias is calculated as a p-value based on the binomial distribution. For hypothesis A, if K is the number of state changes supporting increased A and J is the number of state changes supporting decreased A, then Concordance of A is the probability of making H or more (H = max (J,K)) correct predictions out of (J + K) total predicted and observed changes given that the null probability of the prediction (increase or decrease) matches the observed change (increase or decrease) is 0.5. Richness and Concordance are metrics of the significance of a hypothesis: whether the hypothesis can explain more of the observed changes than would be expected by chance and whether its predictions are more consistent than would be expected by chance.

In the second stage of the analysis, the hypotheses produced for each dataset were filtered for significance and presented in an analysis interface that facilitated the sorting of hypotheses by multiple criteria, comparison of hypotheses between experiments, and the investigation of the literature citations supporting each hypothesis. A hypothesis was considered to be statistically (although not necessarily biologically) significant if it met richness and concordance probability cutoffs of 0.05, and marginally significant if it met richness and concordance probability cutoffs of 0.1. Scientists using this analysis interface selected hypotheses for inclusion in the explanatory networks and eventually the CNM based on criteria including (1) whether the nodes in the hypothesis were causally linked to phenotypes and processes observed in the study, (2) whether the hypothesis node was causally downstream from GSK690693, (3) whether the root node was causally connected to other hypothesis root nodes, and (4) whether the root node itself was an increased or decreased state change. The four mechanisms presented in this report are the networks common to the Causal Network models constructed for each treatment of sensitive cell lines in cell culture and xenografts. A summary overview of the Causal Reasoning methodology is shown in Additional file 13 Figure S5. References for all data in the additional files can be found in Additional file 14 Table S9.

## Competing interests

RK, SJB and CEE are current or former employees of GlaxoSmithKline. DP, MM, ALM, JJL and KE are current or former employees of Genstruct.

## Authors' contributions

RK designed the experiment, SJB and CEE generated the Affymetrix data. EFP generated the phosphoproteomic data, DP, MM, ALM, JJL and KE performed Reverse Causal Reasoning on the data. All authors have read and approved the final manuscript.

## Supplementary Material

Additional file 1 Table S1: Summary of State Changes; table Click here for file

Additional file 2 Table S2: Increased Transcriptional Activity of FOXO1 and FOXO3; table Click here for file

Additional file 3 Table S3: Decreased Myc Activity; table Click here for file

Additional file 4 Figure S1: MYC RNA Levels; bar graph Click here for file

Additional file 5 Table S4: Decreased Transcriptional Activity of MYC In SKOV3 Cell Culture at 2 Hours; table Click here for file

Additional file 6 Figure S2: HBP1, CCNG2, and CDKN1B RNA Levels; bar graphs Click here for file

Additional file 7 Figure S3: MXI1 RNA Levels; bar graph Click here for file

Additional file 8 Table S5: Decreased TFRC Activity; table Click here for file

Additional file 9 Figure S4: TFRC RNA Levels; bar graph Click here for file

Additional File 10 Table S6: Increased Transcriptional Activity of RB1; table Click here for file

Additional file 11 Table S7: Increased CDKN1A Activity; table Click here for file

Additional file 12 Table S8: Decreased Transcriptional Activity of E2F1; table Click here for file

Additional file 13 Figure S5: Overview of Causal Reasoning methodology Click here for file

Additional file 14 Table S9: Supplemental Data References; text Click here for file
