# Supplementary figures and images for: Causal reasoning identifies mechanisms of sensitivity for a novel AKT kinase inhibitor, GSK690693
Source: BMC Genomics. 2010 Jul 6;11:419. doi: 10.1186/1471-2164-11-419 (PMC2996947; doi:10.1186/1471-2164-11-419)

## Slide 1
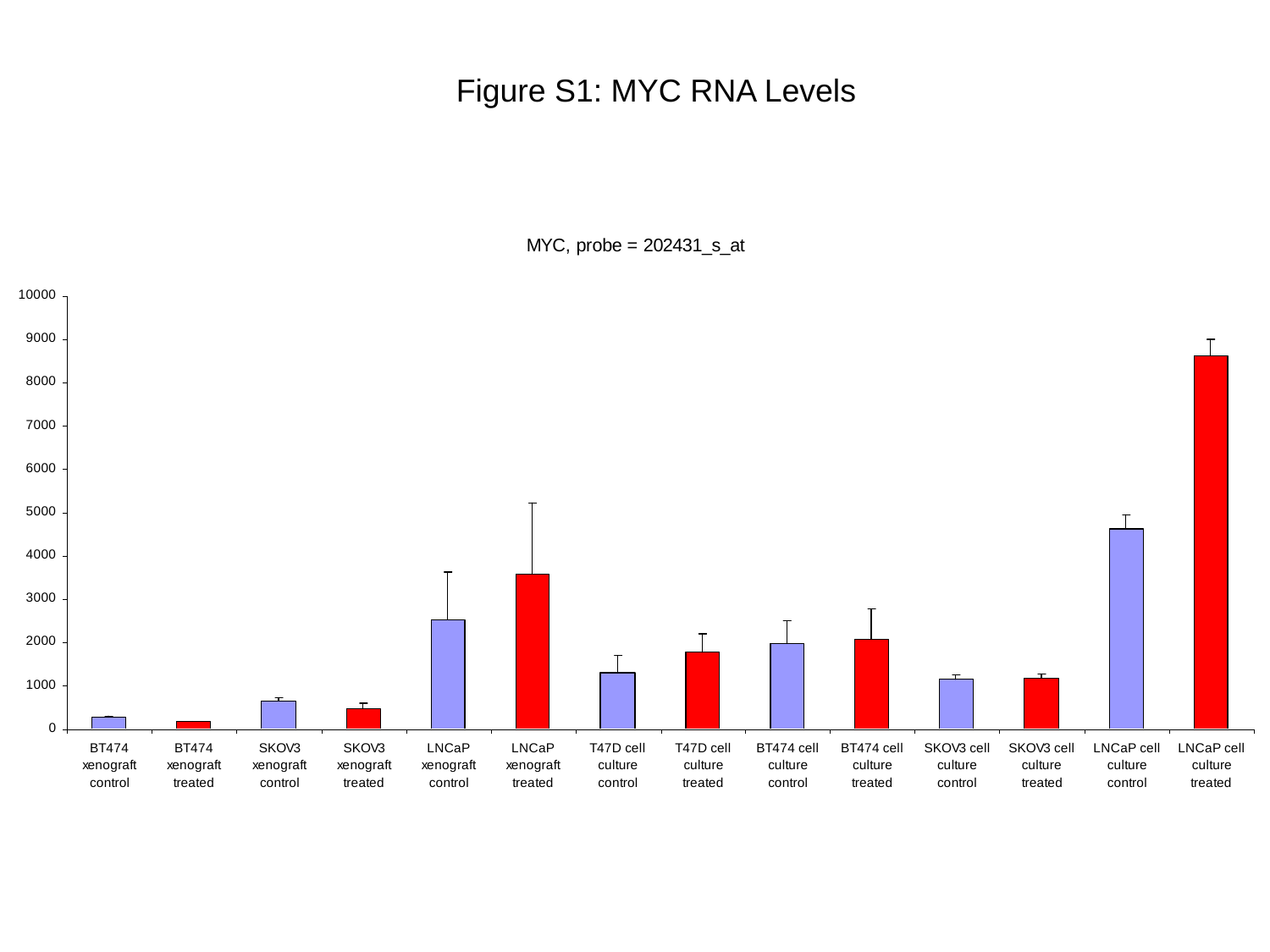

Figure S1: MYC RNA Levels

Supplement: Additional file 4 — Figure S1: MYC RNA Levels; bar graph [file 1471-2164-11-419-S4.PPT]

## Slide 1
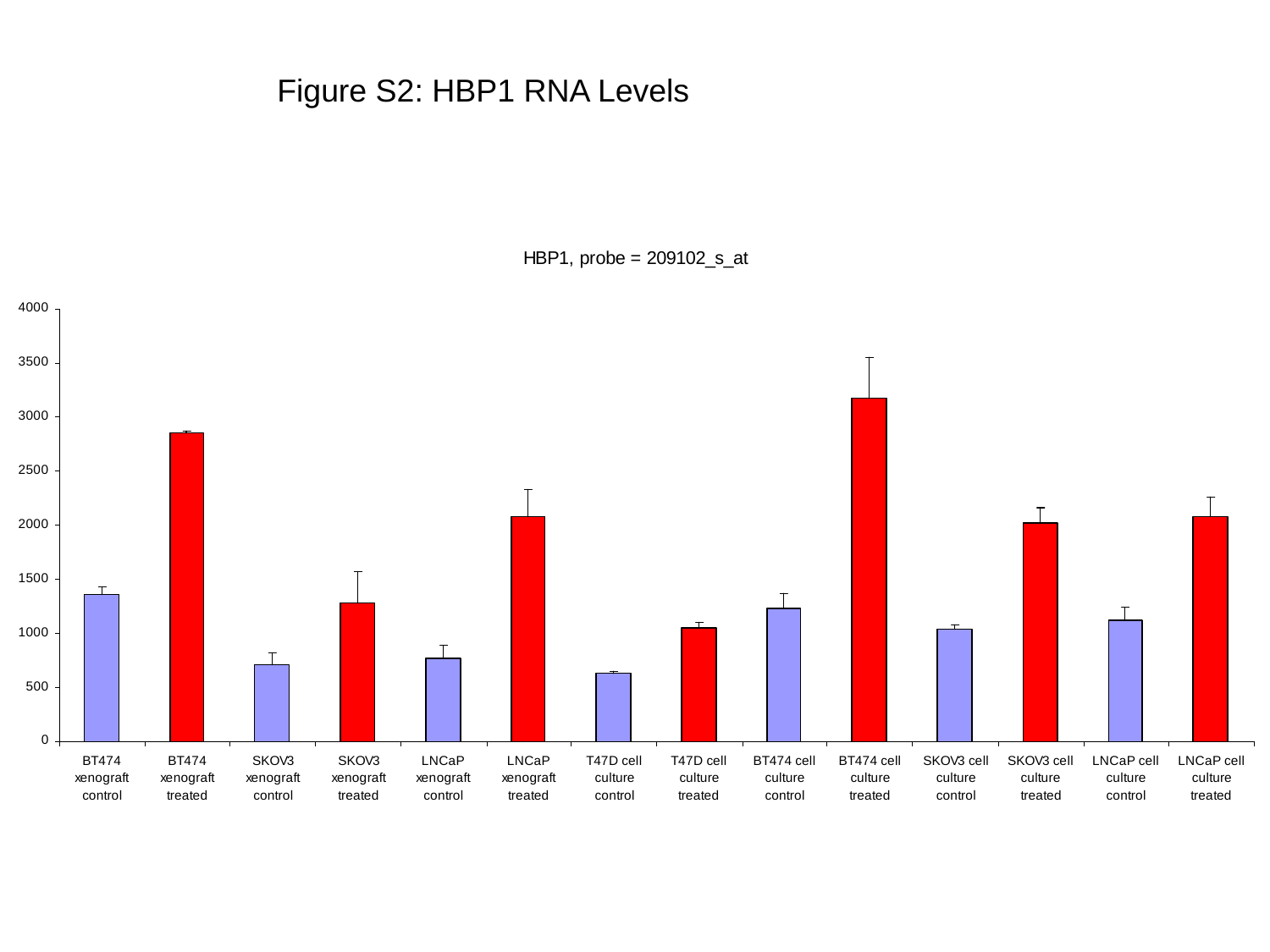

Figure S2: HBP1 RNA Levels

## Slide 2
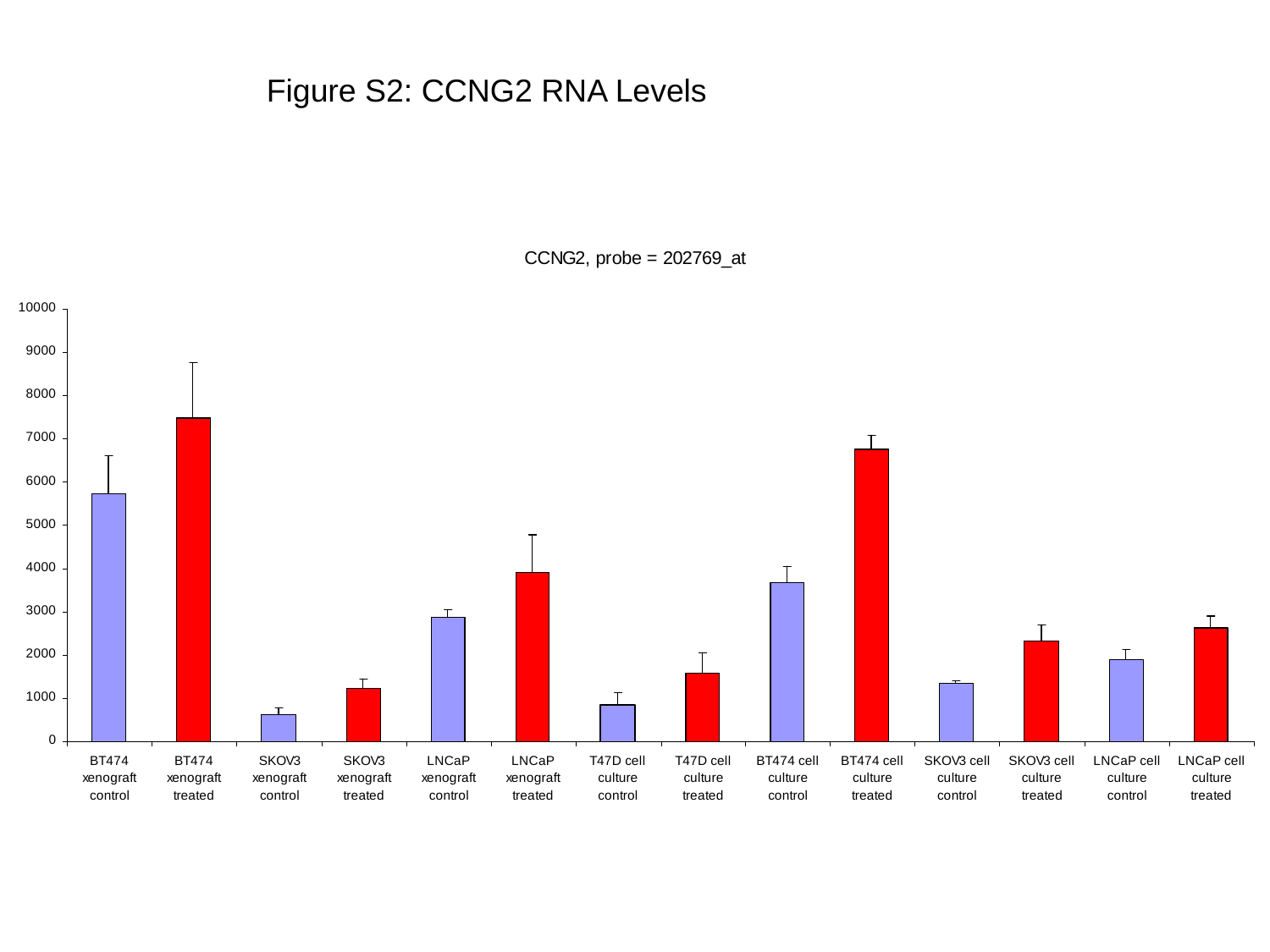

Figure S2: CCNG2 RNA Levels

## Slide 3
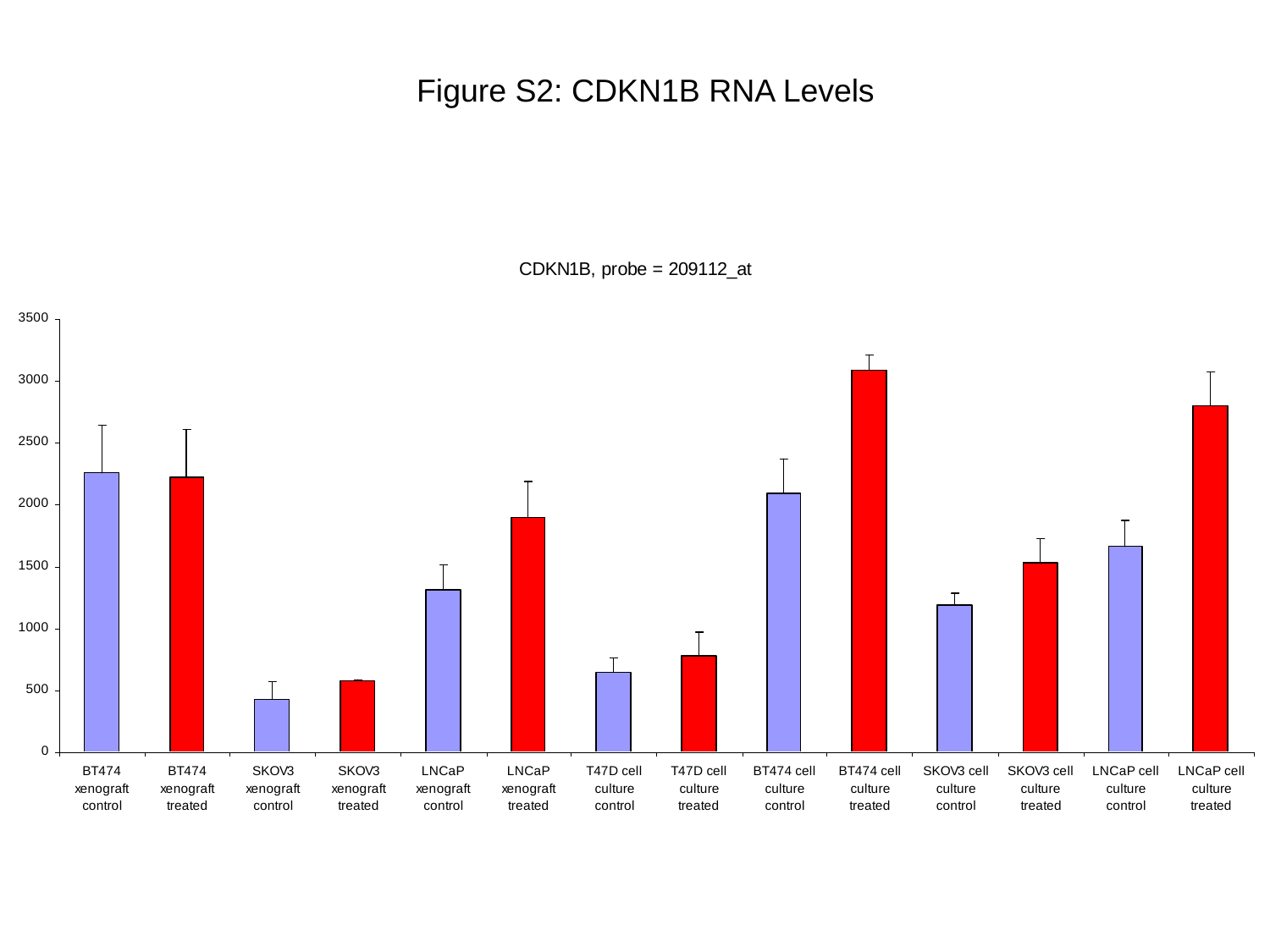

Figure S2: CDKN1B RNA Levels

Supplement: Additional file 6 — Figure S2: HBP1, CCNG2, and CDKN1B RNA Levels; bar graphs [file 1471-2164-11-419-S6.PPT]

## Slide 1
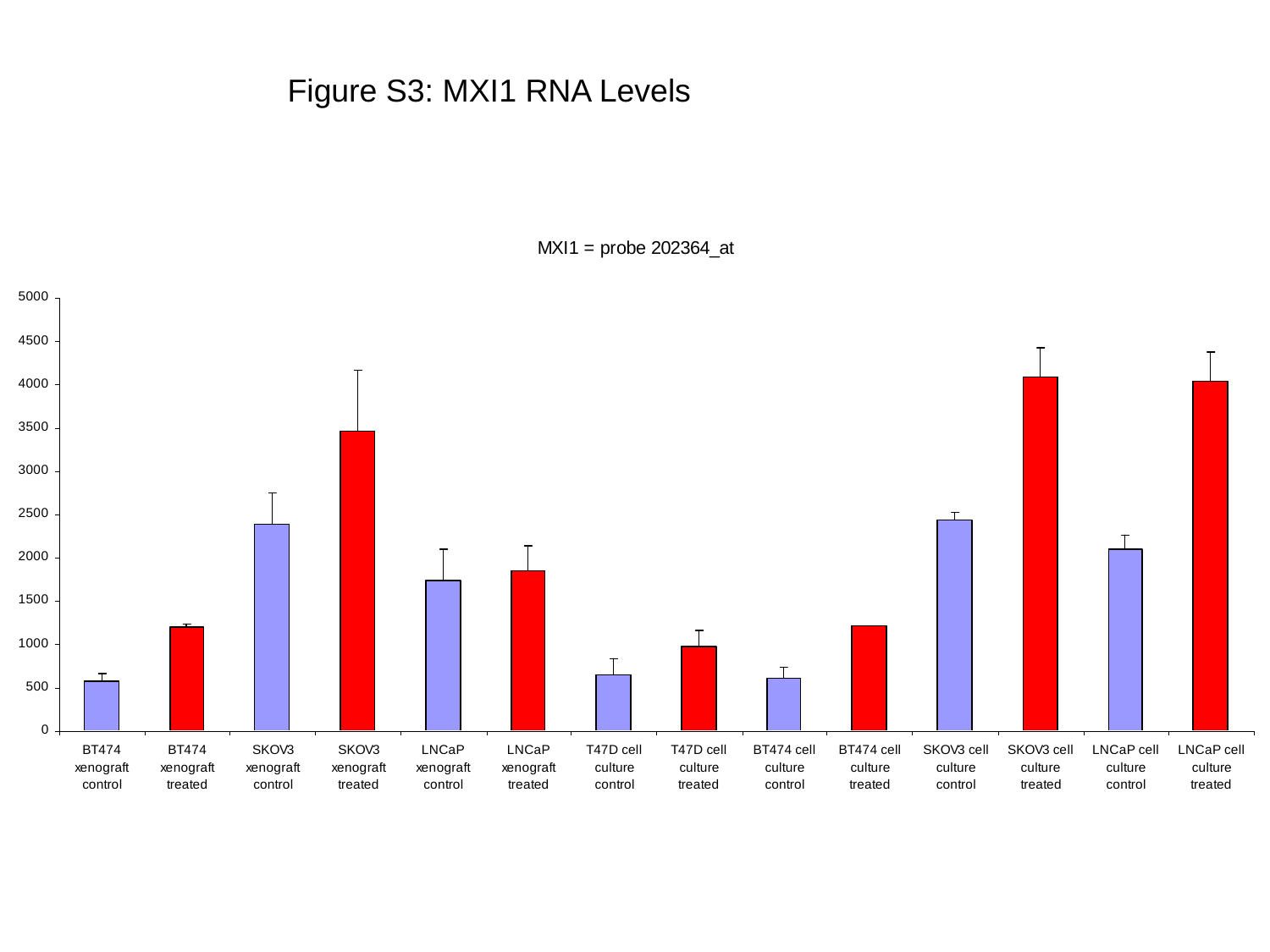

Figure S3: MXI1 RNA Levels

Supplement: Additional file 7 — Figure S3: MXI1 RNA Levels; bar graph [file 1471-2164-11-419-S7.PPT]

## Slide 1
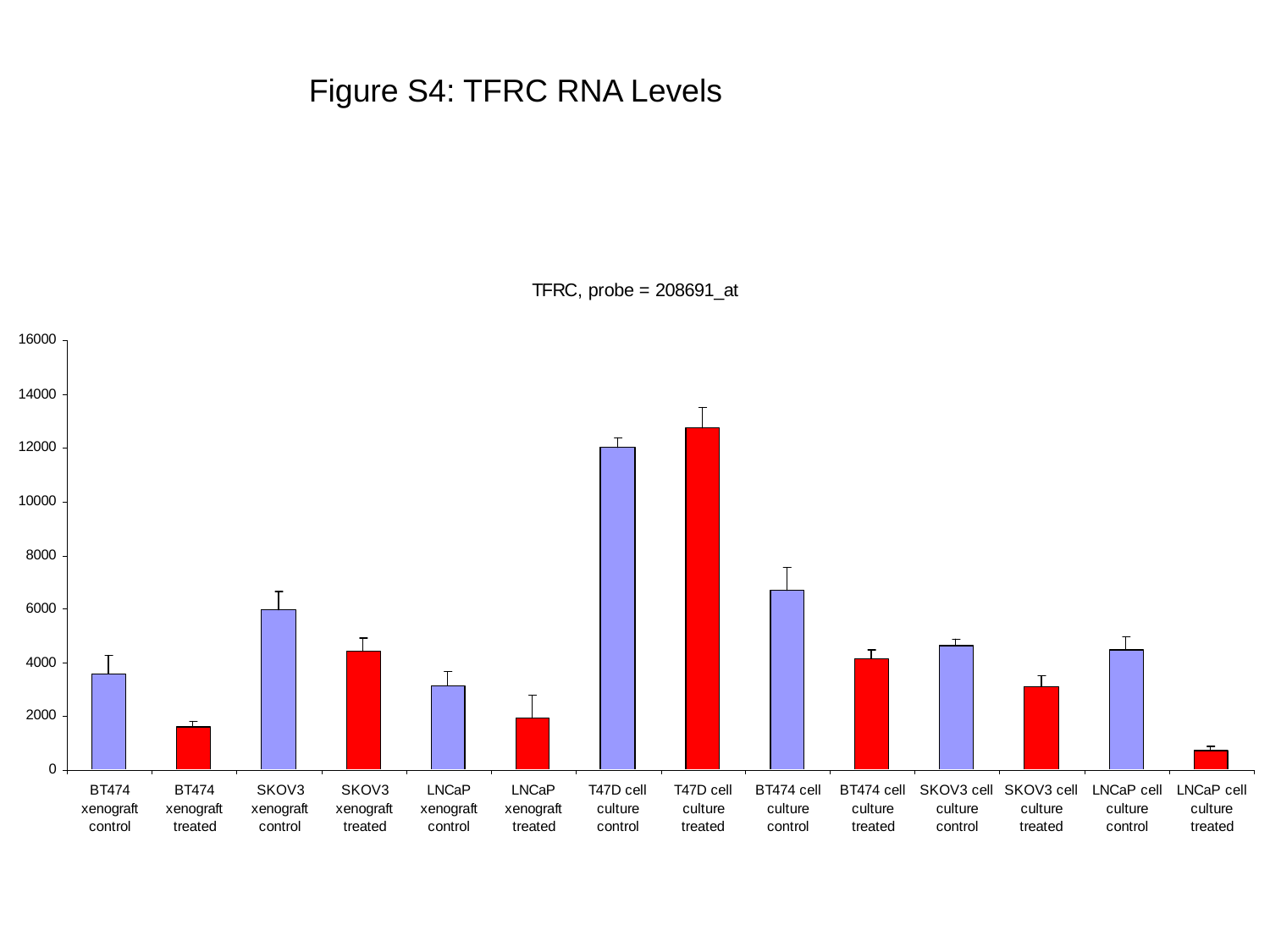

Figure S4: TFRC RNA Levels

Supplement: Additional file 9 — Figure S4: TFRC RNA Levels; bar graph [file 1471-2164-11-419-S9.PPT]
