# Supplementary material for: Causal reasoning identifies mechanisms of sensitivity for a novel AKT kinase inhibitor, GSK690693
Source: BMC Genomics. 2010 Jul 6;11:419. doi: 10.1186/1471-2164-11-419 (PMC2996947; doi:10.1186/1471-2164-11-419)
Supplement: Additional file 13 — Figure S5: Overview of Causal Reasoning methodology [file 1471-2164-11-419-S13.PPT]

## Slide 1
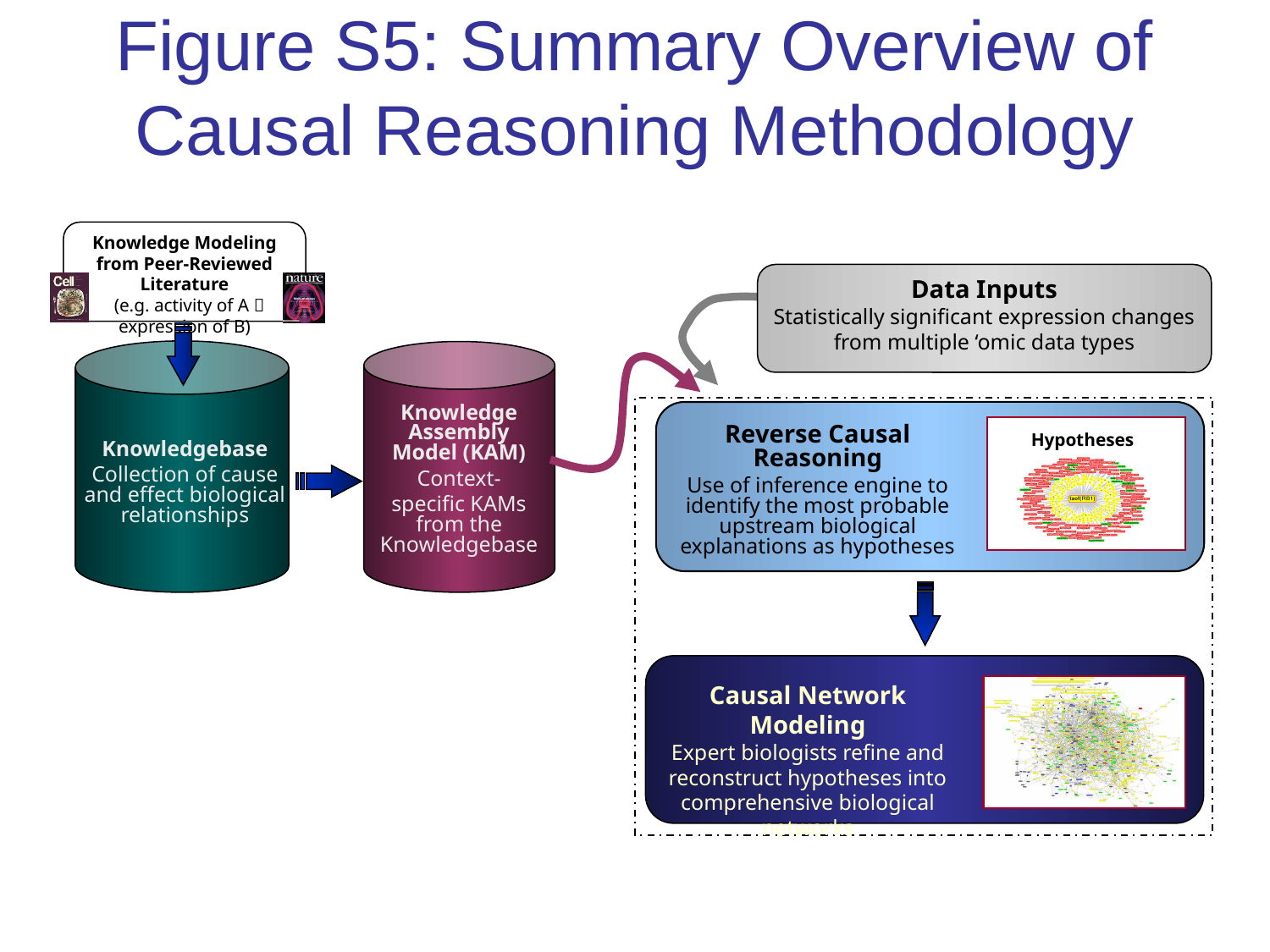

# Figure S5: Summary Overview of Causal Reasoning Methodology
Knowledge Modeling from Peer-Reviewed Literature
 (e.g. activity of A  expression of B)
Data Inputs
Statistically significant expression changes from multiple ‘omic data types
Knowledge Assembly Model (KAM)
Context-
specific KAMs from the Knowledgebase
Reverse Causal Reasoning
Use of inference engine to identify the most probable upstream biological explanations as hypotheses
Hypotheses
Knowledgebase
Collection of cause and effect biological relationships
Causal Network Modeling
Expert biologists refine and reconstruct hypotheses into comprehensive biological networks
