# Supplementary material for: Causal reasoning identifies mechanisms of sensitivity for a novel AKT kinase inhibitor, GSK690693
Source: BMC Genomics. 2010 Jul 6;11:419. doi: 10.1186/1471-2164-11-419 (PMC2996947; doi:10.1186/1471-2164-11-419)
Supplement: Additional file 14 — Table S9: Supplemental Data References; text [file 1471-2164-11-419-S14.DOC]

**Table S9 : Supplemental Data References**

[1]

[2]

[3]

[4]

[5]

[6]

[7]

[8]

[9]

[10]

[11]

[12]

[13]

[14]

[15]

[16]

[17]

[18]

[19]

[20]

[21]

[22]

[23]

[24]

[25]

[26]

[27]

[28]

[29]

[30]

[31]

[32]

[33]

[34]

[35]

[36]

[37]

[38]

[39]

[40]

[41]

[42]

[43]

[44]

[45]

[46]

[47]

[48]

[49]

[50]

[51]

[52]

[53]

[54]

[55]

[56]

[57]

[58]

[59]

[60]

[61]

[62]

[63]

[64]

[65]

[66]

[67]

[68]

[69]

[70]

[71]

[72]

[73]

[74]

[75]

[76]

[77]

[78]

[79]

[80]

[81]

[82]

[83]

[84]

[85]

[86]

[87]

[88]

[89]

[90]

[91]

[92]

[93]

[94]

[95]

[96]

[97]

[98]

[99]

[100]

[101]

[102]

[103]

[104]

[105]

[106]

[107]

[108]

[109]

[110]

[111]

[112]

[113]

[114]

[115]

[116]

[117]

[118]

[119]

[120]

[121]

[122]

[123]

[124]

[125]

[126]

[127]

[128]

[129]

[130]

[131]

[132]

[133]

[134]

[135]

[136]

[137]

[138]

[139]

[140]

[141]

[142]

[143]

[144]

[145]

[146]

[147]

[148]

[149]

[150]

[151]

[152]

[153]

[154]

[155]

[156]

[157]

[158]

[159]

[160]

[161]

[162]

[163]

[164]

[165]

[166]

[167]

[168]

[169]

[170]

[171]

[172]

[173]

[174]

[175]

[176]

[177]

[178]

[179]

[180]

[181]

[182]

[183]

[184]

[185]

[186]

[187]

[188]

[189]

[190]

[191]

[192]

[193]

[194]

[195]

[196]

[197]

[198]

[199]

[200]

[201]

[202]

[203]

[204]

[205]

[206]

[207]

[208]

[209]

[210]

[211]

[212]

[213]

**References for Supplemental Data**

1. Modur V, Nagarajan R, Evers BM, Milbrandt J: **FOXO proteins regulate tumor necrosis factor-related apoptosis inducing ligand expression. Implications for PTEN mutation in prostate cancer.** *J Biol Chem* 2002, **277:**47928-47937.

2. Bauer B, Baier G: **Protein kinase C and AKT/protein kinase B in CD4+ T-lymphocytes: new partners in TCR/CD28 signal integration.** *Mol Immunol* 2002, **38:**1087-1099.

3. Zhang X, Gan L, Pan H, Guo S, He X, Olson ST, Mesecar A, Adam S, Unterman TG: **Phosphorylation of serine 256 suppresses transactivation by FKHR (FOXO1) by multiple mechanisms. Direct and indirect effects on nuclear/cytoplasmic shuttling and DNA binding.** *J Biol Chem* 2002, **277:**45276-45284.

4. Li P, Lee H, Guo S, Unterman TG, Jenster G, Bai W: **AKT-independent protection of prostate cancer cells from apoptosis mediated through complex formation between the androgen receptor and FKHR.** *Mol Cell Biol* 2003, **23:**104-118.

5. Yamamura Y, Lee WL, Inoue K, Ida H, Ito Y: **RUNX3 cooperates with FoxO3a to induce apoptosis in gastric cancer cells.** *J Biol Chem* 2006, **281:**5267-5276.

6. Suhara T, Kim HS, Kirshenbaum LA, Walsh K: **Suppression of Akt signaling induces Fas ligand expression: involvement of caspase and Jun kinase activation in Akt-mediated Fas ligand regulation.** *Mol Cell Biol* 2002, **22:**680-691.

7. Leisser C, Rosenberger G, Maier S, Fuhrmann G, Grusch M, Strasser S, Huettenbrenner S, Fassl S, Polgar D, Krieger S, et al: **Subcellular localisation of Cdc25A determines cell fate.** *Cell Death Differ* 2004, **11:**80-89.

8. Asselin-Labat ML, David M, Biola-Vidamment A, Lecoeuche D, Zennaro MC, Bertoglio J, Pallardy M: **GILZ, a new target for the transcription factor FoxO3, protects T lymphocytes from interleukin-2 withdrawal-induced apoptosis.** *Blood* 2004, **104:**215-223.

9. Reed JC: **Apoptosis-targeted therapies for cancer.** *Cancer Cell* 2003, **3:**17-22.

10. Zhu W, Bijur GN, Styles NA, Li X: **Regulation of FOXO3a by brain-derived neurotrophic factor in differentiated human SH-SY5Y neuroblastoma cells.** *Brain Res Mol Brain Res* 2004, **126:**45-56.

11. Essafi A, Fernandez de Mattos S, Hassen YA, Soeiro I, Mufti GJ, Thomas NS, Medema RH, Lam EW: **Direct transcriptional regulation of Bim by FoxO3a mediates STI571-induced apoptosis in Bcr-Abl-expressing cells.** *Oncogene* 2005, **24:**2317-2329.

12. Sunters A, Fernandez de Mattos S, Stahl M, Brosens JJ, Zoumpoulidou G, Saunders CA, Coffer PJ, Medema RH, Coombes RC, Lam EW: **FoxO3a transcriptional regulation of Bim controls apoptosis in paclitaxel-treated breast cancer cell lines.** *J Biol Chem* 2003, **278:**49795-49805.

13. Scheijen B, Ngo HT, Kang H, Griffin JD: **FLT3 receptors with internal tandem duplications promote cell viability and proliferation by signaling through Foxo proteins.** *Oncogene* 2004, **23:**3338-3349.

14. Bakker WJ, van Dijk TB, Parren-van Amelsvoort M, Kolbus A, Yamamoto K, Steinlein P, Verhaak RG, Mak TW, Beug H, Lowenberg B, von Lindern M: **Differential regulation of Foxo3a target genes in erythropoiesis.** *Mol Cell Biol* 2007, **27:**3839-3854.

15. Bakker WJ, Blazquez-Domingo M, Kolbus A, Besooyen J, Steinlein P, Beug H, Coffer PJ, Lowenberg B, von Lindern M, van Dijk TB: **FoxO3a regulates erythroid differentiation and induces BTG1, an activator of protein arginine methyl transferase 1.** *J Cell Biol* 2004, **164:**175-184.

16. Huang H, Regan KM, Wang F, Wang D, Smith DI, van Deursen JM, Tindall DJ: **Skp2 inhibits FOXO1 in tumor suppression through ubiquitin-mediated degradation.** *Proc Natl Acad Sci U S A* 2005, **102:**1649-1654.

17. Bouchard C, Marquardt J, Bras A, Medema RH, Eilers M: **Myc-induced proliferation and transformation require Akt-mediated phosphorylation of FoxO proteins.** *Embo J* 2004, **23:**2830-2840.

18. Tran H, Brunet A, Grenier JM, Datta SR, Fornace AJ, Jr., DiStefano PS, Chiang LW, Greenberg ME: **DNA repair pathway stimulated by the forkhead transcription factor FOXO3a through the Gadd45 protein.** *Science* 2002, **296:**530-534.

19. Chandramohan V, Jeay S, Pianetti S, Sonenshein GE: **Reciprocal control of Forkhead box O 3a and c-Myc via the phosphatidylinositol 3-kinase pathway coordinately regulates p27Kip1 levels.** *J Immunol* 2004, **172:**5522-5527.

20. Abid MR, Guo S, Minami T, Spokes KC, Ueki K, Skurk C, Walsh K, Aird WC: **Vascular endothelial growth factor activates PI3K/Akt/forkhead signaling in endothelial cells.** *Arterioscler Thromb Vasc Biol* 2004, **24:**294-300.

21. Park KW, Kim DH, You HJ, Sir JJ, Jeon SI, Youn SW, Yang HM, Skurk C, Park YB, Walsh K, Kim HS: **Activated forkhead transcription factor inhibits neointimal hyperplasia after angioplasty through induction of p27.** *Arterioscler Thromb Vasc Biol* 2005, **25:**742-747.

22. Lynch RL, Konicek BW, McNulty AM, Hanna KR, Lewis JE, Neubauer BL, Graff JR: **The progression of LNCaP human prostate cancer cells to androgen independence involves decreased FOXO3a expression and reduced p27KIP1 promoter transactivation.** *Mol Cancer Res* 2005, **3:**163-169.

23. Sandri M, Sandri C, Gilbert A, Skurk C, Calabria E, Picard A, Walsh K, Schiaffino S, Lecker SH, Goldberg AL: **Foxo transcription factors induce the atrophy-related ubiquitin ligase atrogin-1 and cause skeletal muscle atrophy.** *Cell* 2004, **117:**399-412.

24. Sartorelli V, Fulco M: **Molecular and cellular determinants of skeletal muscle atrophy and hypertrophy.** *Sci STKE* 2004, **2004:**re11.

25. Skurk C, Izumiya Y, Maatz H, Razeghi P, Shiojima I, Sandri M, Sato K, Zeng L, Schiekofer S, Pimentel D, et al: **The FOXO3a transcription factor regulates cardiac myocyte size downstream of AKT signaling.** *J Biol Chem* 2005, **280:**20814-20823.

26. Latres E, Amini AR, Amini AA, Griffiths J, Martin FJ, Wei Y, Lin HC, Yancopoulos GD, Glass DJ: **Insulin-like growth factor-1 (IGF-1) inversely regulates atrophy-induced genes via the phosphatidylinositol 3-kinase/Akt/mammalian target of rapamycin (PI3K/Akt/mTOR) pathway.** *J Biol Chem* 2005, **280:**2737-2744.

27. Nadal A, Marrero PF, Haro D: **Down-regulation of the mitochondrial 3-hydroxy-3-methylglutaryl-CoA synthase gene by insulin: the role of the forkhead transcription factor FKHRL1.** *Biochem J* 2002, **366:**289-297.

28. Delpuech O, Griffiths B, East P, Essafi A, Lam EW, Burgering B, Downward J, Schulze A: **Induction of Mxi1-SR alpha by FOXO3a contributes to repression of Myc-dependent gene expression.** *Mol Cell Biol* 2007, **27:**4917-4930.

29. Kwon HS, Huang B, Unterman TG, Harris RA: **Protein kinase B-alpha inhibits human pyruvate dehydrogenase kinase-4 gene induction by dexamethasone through inactivation of FOXO transcription factors.** *Diabetes* 2004, **53:**899-910.

30. Kops GJ, Medema RH, Glassford J, Essers MA, Dijkers PF, Coffer PJ, Lam EW, Burgering BM: **Control of cell cycle exit and entry by protein kinase B-regulated forkhead transcription factors.** *Mol Cell Biol* 2002, **22:**2025-2036.

31. Nemoto S, Fergusson MM, Finkel T: **Nutrient availability regulates SIRT1 through a forkhead-dependent pathway.** *Science* 2004, **306:**2105-2108.

32. Samatar AA, Wang L, Mirza A, Koseoglu S, Liu S, Kumar CC: **Transforming growth factor-beta 2 is a transcriptional target for Akt/protein kinase B via forkhead transcription factor.** *J Biol Chem* 2002, **277:**28118-28126.

33. Yang L, Xie S, Jamaluddin MS, Altuwaijri S, Ni J, Kim E, Chen YT, Hu YC, Wang L, Chuang KH, et al: **Induction of androgen receptor expression by phosphatidylinositol 3-kinase/Akt downstream substrate, FOXO3a, and their roles in apoptosis of LNCaP prostate cancer cells.** *J Biol Chem* 2005, **280:**33558-33565.

34. Ghaffari S, Jagani Z, Kitidis C, Lodish HF, Khosravi-Far R: **Cytokines and BCR-ABL mediate suppression of TRAIL-induced apoptosis through inhibition of forkhead FOXO3a transcription factor.** *Proc Natl Acad Sci U S A* 2003, **100:**6523-6528.

35. Takano M, Lu Z, Goto T, Fusi L, Higham J, Francis J, Withey A, Hardt J, Cloke B, Stavropoulou AV, et al: **Transcriptional cross talk between the forkhead transcription factor forkhead box O1A and the progesterone receptor coordinates cell cycle regulation and differentiation in human endometrial stromal cells.** *Mol Endocrinol* 2007, **21:**2334-2349.

36. Yamada T, Ozaki N, Kato Y, Miura Y, Oiso Y: **Insulin downregulates angiopoietin-like protein 4 mRNA in 3T3-L1 adipocytes.** *Biochem Biophys Res Commun* 2006, **347:**1138-1144.

37. Bois PR, Grosveld GC: **FKHR (FOXO1a) is required for myotube fusion of primary mouse myoblasts.** *Embo J* 2003, **22:**1147-1157.

38. Ramaswamy S, Nakamura N, Sansal I, Bergeron L, Sellers WR: **A novel mechanism of gene regulation and tumor suppression by the transcription factor FKHR.** *Cancer Cell* 2002, **2:**81-91.

39. Abid MR, Shih SC, Otu HH, Spokes KC, Okada Y, Curiel DT, Minami T, Aird WC: **A novel class of vascular endothelial growth factor-responsive genes that require forkhead activity for expression.** *J Biol Chem* 2006, **281:**35544-35553.

40. Christian SL, Lee RL, McLeod SJ, Burgess AE, Li AH, Dang-Lawson M, Lin KB, Gold MR: **Activation of the Rap GTPases in B lymphocytes modulates B cell antigen receptor-induced activation of Akt but has no effect on MAPK activation.** *J Biol Chem* 2003, **278:**41756-41767.

41. Hisahara S, Chiba S, Matsumoto H, Horio Y: **Transcriptional regulation of neuronal genes and its effect on neural functions: NAD-dependent histone deacetylase SIRT1 (Sir2alpha).** *J Pharmacol Sci* 2005, **98:**200-204.

42. Kamei Y, Miura S, Suzuki M, Kai Y, Mizukami J, Taniguchi T, Mochida K, Hata T, Matsuda J, Aburatani H, et al: **Skeletal muscle FOXO1 (FKHR) transgenic mice have less skeletal muscle mass, down-regulated Type I (slow twitch/red muscle) fiber genes, and impaired glycemic control.** *J Biol Chem* 2004, **279:**41114-41123.

43. Buzzio OL, Lu Z, Miller CD, Unterman TG, Kim JJ: **FOXO1A differentially regulates genes of decidualization.** *Endocrinology* 2006, **147:**3870-3876.

44. Guo S, Rena G, Cichy S, He X, Cohen P, Unterman T: **Phosphorylation of serine 256 by protein kinase B disrupts transactivation by FKHR and mediates effects of insulin on insulin-like growth factor-binding protein-1 promoter activity through a conserved insulin response sequence.** *J Biol Chem* 1999, **274:**17184-17192.

45. Daitoku H, Yamagata K, Matsuzaki H, Hatta M, Fukamizu A: **Regulation of PGC-1 promoter activity by protein kinase B and the forkhead transcription factor FKHR.** *Diabetes* 2003, **52:**642-649.

46. Spriet LL, Tunstall RJ, Watt MJ, Mehan KA, Hargreaves M, Cameron-Smith D: **Pyruvate dehydrogenase activation and kinase expression in human skeletal muscle during fasting.** *J Appl Physiol* 2004, **96:**2082-2087.

47. Peng XD, Xu PZ, Chen ML, Hahn-Windgassen A, Skeen J, Jacobs J, Sundararajan D, Chen WS, Crawford SE, Coleman KG, Hay N: **Dwarfism, impaired skin development, skeletal muscle atrophy, delayed bone development, and impeded adipogenesis in mice lacking Akt1 and Akt2.** *Genes Dev* 2003, **17:**1352-1365.

48. Armoni M, Harel C, Karni S, Chen H, Bar-Yoseph F, Ver MR, Quon MJ, Karnieli E: **FOXO1 represses peroxisome proliferator-activated receptor-gamma1 and -gamma2 gene promoters in primary adipocytes. A novel paradigm to increase insulin sensitivity.** *J Biol Chem* 2006, **281:**19881-19891.

49. Naimi M, Gautier N, Chaussade C, Valverde AM, Accili D, Van Obberghen E: **Nuclear forkhead box O1 controls and integrates key signaling pathways in hepatocytes.** *Endocrinology* 2007, **148:**2424-2434.

50. Gebhardt A, Frye M, Herold S, Benitah SA, Braun K, Samans B, Watt FM, Elsasser HP, Eilers M: **Myc regulates keratinocyte adhesion and differentiation via complex formation with Miz1.** *J Cell Biol* 2006, **172:**139-149.

51. McConnell MJ, Chevallier N, Berkofsky-Fessler W, Giltnane JM, Malani RB, Staudt LM, Licht JD: **Growth suppression by acute promyelocytic leukemia-associated protein PLZF is mediated by repression of c-myc expression.** *Mol Cell Biol* 2003, **23:**9375-9388.

52. Schuldiner O, Shor S, Benvenisty N: **A computerized database-scan to identify c-MYC targets.** *Gene* 2002, **292:**91-99.

53. Mao DY, Watson JD, Yan PS, Barsyte-Lovejoy D, Khosravi F, Wong WW, Farnham PJ, Huang TH, Penn LZ: **Analysis of Myc bound loci identified by CpG island arrays shows that Max is essential for Myc-dependent repression.** *Curr Biol* 2003, **13:**882-886.

54. Frye M, Gardner C, Li ER, Arnold I, Watt FM: **Evidence that Myc activation depletes the epidermal stem cell compartment by modulating adhesive interactions with the local microenvironment.** *Development* 2003, **130:**2793-2808.

55. O'Connell BC, Cheung AF, Simkevich CP, Tam W, Ren X, Mateyak MK, Sedivy JM: **A large scale genetic analysis of c-Myc-regulated gene expression patterns.** *J Biol Chem* 2003, **278:**12563-12573.

56. Menssen A, Hermeking H: **Characterization of the c-MYC-regulated transcriptome by SAGE: identification and analysis of c-MYC target genes.** *Proc Natl Acad Sci U S A* 2002, **99:**6274-6279.

57. Marinkovic D, Marinkovic T, Kokai E, Barth T, Moller P, Wirth T: **Identification of novel Myc target genes with a potential role in lymphomagenesis.** *Nucleic Acids Res* 2004, **32:**5368-5378.

58. Coller HA, Grandori C, Tamayo P, Colbert T, Lander ES, Eisenman RN, Golub TR: **Expression analysis with oligonucleotide microarrays reveals that MYC regulates genes involved in growth, cell cycle, signaling, and adhesion.** *Proc Natl Acad Sci U S A* 2000, **97:**3260-3265.

59. Watson JD, Oster SK, Shago M, Khosravi F, Penn LZ: **Identifying genes regulated in a Myc-dependent manner.** *J Biol Chem* 2002, **277:**36921-36930.

60. Schlosser I, Holzel M, Murnseer M, Burtscher H, Weidle UH, Eick D: **A role for c-Myc in the regulation of ribosomal RNA processing.** *Nucleic Acids Res* 2003, **31:**6148-6156.

61. Guney I, Wu S, Sedivy JM: **Reduced c-Myc signaling triggers telomere-independent senescence by regulating Bmi-1 and p16(INK4a).** *Proc Natl Acad Sci U S A* 2006, **103:**3645-3650.

62. Guo WJ, Datta S, Band V, Dimri GP: **Mel-18, a polycomb group protein, regulates cell proliferation and senescence via transcriptional repression of Bmi-1 and c-Myc oncoproteins.** *Mol Biol Cell* 2007, **18:**536-546.

63. Lawlor ER, Soucek L, Brown-Swigart L, Shchors K, Bialucha CU, Evan GI: **Reversible kinetic analysis of Myc targets in vivo provides novel insights into Myc-mediated tumorigenesis.** *Cancer Res* 2006, **66:**4591-4601.

64. Xie Z, Zeng X, Waldman T, Glazer RI: **Transformation of mammary epithelial cells by 3-phosphoinositide- dependent protein kinase-1 activates beta-catenin and c-Myc, and down-regulates caveolin-1.** *Cancer Res* 2003, **63:**5370-5375.

65. Park DS, Razani B, Lasorella A, Schreiber-Agus N, Pestell RG, Iavarone A, Lisanti MP: **Evidence that Myc isoforms transcriptionally repress caveolin-1 gene expression via an INR-dependent mechanism.** *Biochemistry* 2001, **40:**3354-3362.

66. Timme TL, Goltsov A, Tahir S, Li L, Wang J, Ren C, Johnston RN, Thompson TC: **Caveolin-1 is regulated by c-myc and suppresses c-myc-induced apoptosis.** *Oncogene* 2000, **19:**3256-3265.

67. Domashenko AD, Latham KE, Hatton KS: **Expression of myc-family, myc-interacting, and myc-target genes during preimplantation mouse development.** *Mol Reprod Dev* 1997, **47:**57-65.

68. Jansen-Durr P, Meichle A, Steiner P, Pagano M, Finke K, Botz J, Wessbecher J, Draetta G, Eilers M: **Differential modulation of cyclin gene expression by MYC.** *Proc Natl Acad Sci U S A* 1993, **90:**3685-3689.

69. Perez-Roger I, Solomon DL, Sewing A, Land H: **Myc activation of cyclin E/Cdk2 kinase involves induction of cyclin E gene transcription and inhibition of p27(Kip1) binding to newly formed complexes.** *Oncogene* 1997, **14:**2373-2381.

70. Yin XY, Grove L, Datta NS, Katula K, Long MW, Prochownik EV: **Inverse regulation of cyclin B1 by c-Myc and p53 and induction of tetraploidy by cyclin B1 overexpression.** *Cancer Res* 2001, **61:**6487-6493.

71. Perez-Roger I, Kim SH, Griffiths B, Sewing A, Land H: **Cyclins D1 and D2 mediate myc-induced proliferation via sequestration of p27(Kip1) and p21(Cip1).** *Embo J* 1999, **18:**5310-5320.

72. Tamir A, Petrocelli T, Stetler K, Chu W, Howard J, Croix BS, Slingerland J, Ben-David Y: **Stem cell factor inhibits erythroid differentiation by modulating the activity of G1-cyclin-dependent kinase complexes: a role for p27 in erythroid differentiation coupled G1 arrest.** *Cell Growth Differ* 2000, **11:**269-277.

73. Bush A, Mateyak M, Dugan K, Obaya A, Adachi S, Sedivy J, Cole M: **c-myc null cells misregulate cad and gadd45 but not other proposed c-Myc targets.** *Genes Dev* 1998, **12:**3797-3802.

74. Ben-Yosef T, Yanuka O, Halle D, Benvenisty N: **Involvement of Myc targets in c-myc and N-myc induced human tumors.** *Oncogene* 1998, **17:**165-171.

75. Barre B, Vigneron A, Coqueret O: **The STAT3 transcription factor is a target for the Myc and riboblastoma proteins on the Cdc25A promoter.** *J Biol Chem* 2005, **280:**15673-15681.

76. Galaktionov K, Chen X, Beach D: **Cdc25 cell-cycle phosphatase as a target of c-myc.** *Nature* 1996, **382:**511-517.

77. Bouchard C, Dittrich O, Kiermaier A, Dohmann K, Menkel A, Eilers M, Luscher B: **Regulation of cyclin D2 gene expression by the Myc/Max/Mad network: Myc-dependent TRRAP recruitment and histone acetylation at the cyclin D2 promoter.** *Genes Dev* 2001, **15:**2042-2047.

78. Ravanko K, Jarvinen K, Paasinen-Sohns A, Holtta E: **Loss of p27Kip1 from cyclin E/cyclin-dependent kinase (CDK) 2 but not from cyclin D1/CDK4 complexes in cells transformed by polyamine biosynthetic enzymes.** *Cancer Res* 2000, **60:**5244-5253.

79. Philipp A, Schneider A, Vasrik I, Finke K, Xiong Y, Beach D, Alitalo K, Eilers M: **Repression of cyclin D1: a novel function of MYC.** *Mol Cell Biol* 1994, **14:**4032-4043.

80. Obaya AJ, Kotenko I, Cole MD, Sedivy JM: **The proto-oncogene c-myc acts through the cyclin-dependent kinase (Cdk) inhibitor p27(Kip1) to facilitate the activation of Cdk4/6 and early G(1) phase progression.** *J Biol Chem* 2002, **277:**31263-31269.

81. Bouchard C, Thieke K, Maier A, Saffrich R, Hanley-Hyde J, Ansorge W, Reed S, Sicinski P, Bartek J, Eilers M: **Direct induction of cyclin D2 by Myc contributes to cell cycle progression and sequestration of p27.** *Embo J* 1999, **18:**5321-5333.

82. Swarbrick A, Akerfeldt MC, Lee CS, Sergio CM, Caldon CE, Hunter LJ, Sutherland RL, Musgrove EA: **Regulation of cyclin expression and cell cycle progression in breast epithelial cells by the helix-loop-helix protein Id1.** *Oncogene* 2005, **24:**381-389.

83. Barre B, Vigneron A, Perkins N, Roninson IB, Gamelin E, Coqueret O: **The STAT3 oncogene as a predictive marker of drug resistance.** *Trends Mol Med* 2007, **13:**4-11.

84. O'Hagan RC, Schreiber-Agus N, Chen K, David G, Engelman JA, Schwab R, Alland L, Thomson C, Ronning DR, Sacchettini JC, et al: **Gene-target recognition among members of the myc superfamily and implications for oncogenesis.** *Nat Genet* 2000, **24:**113-119.

85. Mu ZM, Yin XY, Prochownik EV: **Pag, a putative tumor suppressor, interacts with the Myc Box II domain of c-Myc and selectively alters its biological function and target gene expression.** *J Biol Chem* 2002, **277:**43175-43184.

86. Mochizuki T, Kitanaka C, Noguchi K, Muramatsu T, Asai A, Kuchino Y: **Physical and functional interactions between Pim-1 kinase and Cdc25A phosphatase. Implications for the Pim-1-mediated activation of the c-Myc signaling pathway.** *J Biol Chem* 1999, **274:**18659-18666.

87. Prescott JE, Osthus RC, Lee LA, Lewis BC, Shim H, Barrett JF, Guo Q, Hawkins AL, Griffin CA, Dang CV: **A novel c-Myc-responsive gene, JPO1, participates in neoplastic transformation.** *J Biol Chem* 2001, **276:**48276-48284.

88. Huang A, Ho CS, Ponzielli R, Barsyte-Lovejoy D, Bouffet E, Picard D, Hawkins CE, Penn LZ: **Identification of a novel c-Myc protein interactor, JPO2, with transforming activity in medulloblastoma cells.** *Cancer Res* 2005, **65:**5607-5619.

89. Kim YH, Buchholz MA, Chrest FJ, Nordin AA: **Up-regulation of c-myc induces the gene expression of the murine homologues of p34cdc2 and cyclin-dependent kinase-2 in T lymphocytes.** *J Immunol* 1994, **152:**4328-4335.

90. Hermeking H, Rago C, Schuhmacher M, Li Q, Barrett JF, Obaya AJ, O'Connell BC, Mateyak MK, Tam W, Kohlhuber F, et al: **Identification of CDK4 as a target of c-MYC.** *Proc Natl Acad Sci U S A* 2000, **97:**2229-2234.

91. Hermeking H: **The MYC oncogene as a cancer drug target.** *Curr Cancer Drug Targets* 2003, **3:**163-175.

92. Claassen GF, Hann SR: **A role for transcriptional repression of p21CIP1 by c-Myc in overcoming transforming growth factor beta -induced cell-cycle arrest.** *Proc Natl Acad Sci U S A* 2000, **97:**9498-9503.

93. Vigneron A, Roninson IB, Gamelin E, Coqueret O: **Src inhibits adriamycin-induced senescence and G2 checkpoint arrest by blocking the induction of p21waf1.** *Cancer Res* 2005, **65:**8927-8935.

94. Ceballos E, Munoz-Alonso MJ, Berwanger B, Acosta JC, Hernandez R, Krause M, Hartmann O, Eilers M, Leon J: **Inhibitory effect of c-Myc on p53-induced apoptosis in leukemia cells. Microarray analysis reveals defective induction of p53 target genes and upregulation of chaperone genes.** *Oncogene* 2005, **24:**4559-4571.

95. Yang W, Shen J, Wu M, Arsura M, FitzGerald M, Suldan Z, Kim DW, Hofmann CS, Pianetti S, Romieu-Mourez R, et al: **Repression of transcription of the p27(Kip1) cyclin-dependent kinase inhibitor gene by c-Myc.** *Oncogene* 2001, **20:**1688-1702.

96. Yang W, Bancroft L, Nicholas C, Lozonschi I, Augenlicht LH: **Targeted inactivation of p27kip1 is sufficient for large and small intestinal tumorigenesis in the mouse, which can be augmented by a Western-style high-risk diet.** *Cancer Res* 2003, **63:**4990-4996.

97. Gartel AL, Ye X, Goufman E, Shianov P, Hay N, Najmabadi F, Tyner AL: **Myc represses the p21(WAF1/CIP1) promoter and interacts with Sp1/Sp3.** *Proc Natl Acad Sci U S A* 2001, **98:**4510-4515.

98. O'Hagan RC, Ohh M, David G, de Alboran IM, Alt FW, Kaelin WG, Jr., DePinho RA: **Myc-enhanced expression of Cul1 promotes ubiquitin-dependent proteolysis and cell cycle progression.** *Genes Dev* 2000, **14:**2185-2191.

99. van de Wetering M, Sancho E, Verweij C, de Lau W, Oving I, Hurlstone A, van der Horn K, Batlle E, Coudreuse D, Haramis AP, et al: **The beta-catenin/TCF-4 complex imposes a crypt progenitor phenotype on colorectal cancer cells.** *Cell* 2002, **111:**241-250.

100. Yang SZ, Kohno N, Yokoyama A, Kondo K, Hamada H, Hiwada K: **Decreased E-cadherin augments beta-catenin nuclear localization: studies in breast cancer cell lines.** *Int J Oncol* 2001, **18:**541-548.

101. Adhikary S, Peukert K, Karsunky H, Beuger V, Lutz W, Elsasser HP, Moroy T, Eilers M: **Miz1 is required for early embryonic development during gastrulation.** *Mol Cell Biol* 2003, **23:**7648-7657.

102. Seoane J, Pouponnot C, Staller P, Schader M, Eilers M, Massague J: **TGFbeta influences Myc, Miz-1 and Smad to control the CDK inhibitor p15INK4b.** *Nat Cell Biol* 2001, **3:**400-408.

103. Staller P, Peukert K, Kiermaier A, Seoane J, Lukas J, Karsunky H, Moroy T, Bartek J, Massague J, Hanel F, Eilers M: **Repression of p15INK4b expression by Myc through association with Miz-1.** *Nat Cell Biol* 2001, **3:**392-399.

104. Antonson P, Pray MG, Jacobsson A, Xanthopoulos KG: **Myc inhibits CCAAT/enhancer-binding protein alpha-gene expression in HIB-1B hibernoma cells through interactions with the core promoter region.** *Eur J Biochem* 1995, **232:**397-403.

105. Kolchanov NA, Ignatieva EV, Ananko EA, Podkolodnaya OA, Stepanenko IL, Merkulova TI, Pozdnyakov MA, Podkolodny NL, Naumochkin AN, Romashchenko AG: **Transcription Regulatory Regions Database (TRRD): its status in 2002.** *Nucleic Acids Res* 2002, **30:**312-317.

106. Li LH, Nerlov C, Prendergast G, MacGregor D, Ziff EB: **c-Myc represses transcription in vivo by a novel mechanism dependent on the initiator element and Myc box II.** *Embo J* 1994, **13:**4070-4079.

107. Thomas-Tikhonenko A, Viard-Leveugle I, Dews M, Wehrli P, Sevignani C, Yu D, Ricci S, el-Deiry W, Aronow B, Kaya G, et al: **Myc-transformed epithelial cells down-regulate clusterin, which inhibits their growth in vitro and carcinogenesis in vivo.** *Cancer Res* 2004, **64:**3126-3136.

108. Li F, Wang Y, Zeller KI, Potter JJ, Wonsey DR, O'Donnell KA, Kim JW, Yustein JT, Lee LA, Dang CV: **Myc stimulates nuclearly encoded mitochondrial genes and mitochondrial biogenesis.** *Mol Cell Biol* 2005, **25:**6225-6234.

109. Morrish F, Giedt C, Hockenbery D: **c-MYC apoptotic function is mediated by NRF-1 target genes.** *Genes Dev* 2003, **17:**240-255.

110. Chen C, Nussenzweig A, Guo M, Kim D, Li GC, Ling CC: **Down-regulation of gadd153 by c-myc in rat fibroblasts and its effect on cell growth and radiation-induced apoptosis.** *Oncogene* 1996, **13:**1659-1665.

111. Amundson SA, Zhan Q, Penn LZ, Fornace AJ, Jr.: **Myc suppresses induction of the growth arrest genes gadd34, gadd45, and gadd153 by DNA-damaging agents.** *Oncogene* 1998, **17:**2149-2154.

112. Grandori C, Mac J, Siebelt F, Ayer DE, Eisenman RN: **Myc-Max heterodimers activate a DEAD box gene and interact with multiple E box-related sites in vivo.** *Embo J* 1996, **15:**4344-4357.

113. Zhu K, Henning D, Valdez B, Busch H: **Human RNA helicase II/Gu gene: genomic organization and promoter analysis.** *Biochem Biophys Res Commun* 2001, **281:**1006-1011.

114. Dang CV, Resar LM, Emison E, Kim S, Li Q, Prescott JE, Wonsey D, Zeller K: **Function of the c-Myc oncogenic transcription factor.** *Exp Cell Res* 1999, **253:**63-77.

115. Mai S, Jalava A: **c-Myc binds to 5' flanking sequence motifs of the dihydrofolate reductase gene in cellular extracts: role in proliferation.** *Nucleic Acids Res* 1994, **22:**2264-2273.

116. Cowling VH, D'Cruz CM, Chodosh LA, Cole MD: **c-Myc transforms human mammary epithelial cells through repression of the Wnt inhibitors DKK1 and SFRP1.** *Mol Cell Biol* 2007, **27:**5135-5146.

117. O'Donnell KA, Wentzel EA, Zeller KI, Dang CV, Mendell JT: **c-Myc-regulated microRNAs modulate E2F1 expression.** *Nature* 2005, **435:**839-843.

118. Leone G, DeGregori J, Sears R, Jakoi L, Nevins JR: **Myc and Ras collaborate in inducing accumulation of active cyclin E/Cdk2 and E2F.** *Nature* 1997, **387:**422-426.

119. Sears R, Ohtani K, Nevins JR: **Identification of positively and negatively acting elements regulating expression of the E2F2 gene in response to cell growth signals.** *Mol Cell Biol* 1997, **17:**5227-5235.

120. Leone G, Sears R, Huang E, Rempel R, Nuckolls F, Park CH, Giangrande P, Wu L, Saavedra HI, Field SJ, et al: **Myc requires distinct E2F activities to induce S phase and apoptosis.** *Mol Cell* 2001, **8:**105-113.

121. Rosenwald IB, Rhoads DB, Callanan LD, Isselbacher KJ, Schmidt EV: **Increased expression of eukaryotic translation initiation factors eIF-4E and eIF-2 alpha in response to growth induction by c-myc.** *Proc Natl Acad Sci U S A* 1993, **90:**6175-6178.

122. Jones RM, Branda J, Johnston KA, Polymenis M, Gadd M, Rustgi A, Callanan L, Schmidt EV: **An essential E box in the promoter of the gene encoding the mRNA cap-binding protein (eukaryotic initiation factor 4E) is a target for activation by c-myc.** *Mol Cell Biol* 1996, **16:**4754-4764.

123. Hashimoto K, Nakagawa Y, Morikawa H, Niki M, Egashira Y, Hirata I, Katsu K, Akao Y: **Co-overexpression of DEAD box protein rck/p54 and c-myc protein in human colorectal adenomas and the relevance of their expression in cultured cell lines.** *Carcinogenesis* 2001, **22:**1965-1970.

124. Zhu N, Gu L, Findley HW, Zhou M: **Transcriptional repression of the eukaryotic initiation factor 4E gene by wild type p53.** *Biochem Biophys Res Commun* 2005, **335:**1272-1279.

125. Kim S, Li Q, Dang CV, Lee LA: **Induction of ribosomal genes and hepatocyte hypertrophy by adenovirus-mediated expression of c-Myc in vivo.** *Proc Natl Acad Sci U S A* 2000, **97:**11198-11202.

126. Gartner A, Staiger V: **Neurotrophin secretion from hippocampal neurons evoked by long-term-potentiation-inducing electrical stimulation patterns.** *Proc Natl Acad Sci U S A* 2002, **99:**6386-6391.

127. Osthus RC, Shim H, Kim S, Li Q, Reddy R, Mukherjee M, Xu Y, Wonsey D, Lee LA, Dang CV: **Deregulation of glucose transporter 1 and glycolytic gene expression by c-Myc.** *J Biol Chem* 2000, **275:**21797-21800.

128. Suen TC, Hung MC: **c-myc reverses neu-induced transformed morphology by transcriptional repression.** *Mol Cell Biol* 1991, **11:**354-362.

129. Wu KJ, Polack A, Dalla-Favera R: **Coordinated regulation of iron-controlling genes, H-ferritin and IRP2, by c-MYC.** *Science* 1999, **283:**676-679.

130. Lewis BC, Shim H, Li Q, Wu CS, Lee LA, Maity A, Dang CV: **Identification of putative c-Myc-responsive genes: characterization of rcl, a novel growth-related gene.** *Mol Cell Biol* 1997, **17:**4967-4978.

131. Wood LJ, Mukherjee M, Dolde CE, Xu Y, Maher JF, Bunton TE, Williams JB, Resar LM: **HMG-I/Y, a new c-Myc target gene and potential oncogene.** *Mol Cell Biol* 2000, **20:**5490-5502.

132. Iizuka N, Tsunedomi R, Tamesa T, Okada T, Sakamoto K, Hamaguchi T, Yamada-Okabe H, Miyamoto T, Uchimura S, Hamamoto Y, Oka M: **Involvement of c-myc-regulated genes in hepatocellular carcinoma related to genotype-C hepatitis B virus.** *J Cancer Res Clin Oncol* 2006, **132:**473-481.

133. Kaddurah-Daouk R, Greene JM, Baldwin AS, Jr., Kingston RE: **Activation and repression of mammalian gene expression by the c-myc protein.** *Genes Dev* 1987, **1:**347-357.

134. Kingston RE, Baldwin AS, Jr., Sharp PA: **Regulation of heat shock protein 70 gene expression by c-myc.** *Nature* 1984, **312:**280-282.

135. Ahn SG, Jeong SY, Rhim H, Kim IK: **The role of c-Myc and heat shock protein 70 in human hepatocarcinoma Hep3B cells during apoptosis induced by prostaglandin A2/Delta12-prostaglandin J2.** *Biochim Biophys Acta* 1998, **1448:**115-125.

136. Taira T, Negishi Y, Kihara F, Iguchi-Ariga SM, Ariga H: **c-myc protein complex binds to two sites in human hsp70 promoter region.** *Biochim Biophys Acta* 1992, **1130:**166-174.

137. Frank SR, Schroeder M, Fernandez P, Taubert S, Amati B: **Binding of c-Myc to chromatin mediates mitogen-induced acetylation of histone H4 and gene activation.** *Genes Dev* 2001, **15:**2069-2082.

138. Breit S, Ashman K, Wilting J, Rossler J, Hatzi E, Fotsis T, Schweigerer L: **The N-myc oncogene in human neuroblastoma cells: down-regulation of an angiogenesis inhibitor identified as activin A.** *Cancer Res* 2000, **60:**4596-4601.

139. Shim H, Dolde C, Lewis BC, Wu CS, Dang G, Jungmann RA, Dalla-Favera R, Dang CV: **c-Myc transactivation of LDH-A: implications for tumor metabolism and growth.** *Proc Natl Acad Sci U S A* 1997, **94:**6658-6663.

140. Janssen E, de Groof A, Wijers M, Fransen J, Dzeja PP, Terzic A, Wieringa B: **Adenylate kinase 1 deficiency induces molecular and structural adaptations to support muscle energy metabolism.** *J Biol Chem* 2003, **278:**12937-12945.

141. Schnittger A, Schobinger U, Bouyer D, Weinl C, Stierhof YD, Hulskamp M: **Ectopic D-type cyclin expression induces not only DNA replication but also cell division in Arabidopsis trichomes.** *Proc Natl Acad Sci U S A* 2002, **99:**6410-6415.

142. Tsuneoka M, Koda Y, Soejima M, Teye K, Kimura H: **A novel myc target gene, mina53, that is involved in cell proliferation.** *J Biol Chem* 2002, **277:**35450-35459.

143. Perini G, Diolaiti D, Porro A, Della Valle G: **In vivo transcriptional regulation of N-Myc target genes is controlled by E-box methylation.** *Proc Natl Acad Sci U S A* 2005, **102:**12117-12122.

144. Koshiji M, To KK, Hammer S, Kumamoto K, Harris AL, Modrich P, Huang LE: **HIF-1alpha induces genetic instability by transcriptionally downregulating MutSalpha expression.** *Mol Cell* 2005, **17:**793-803.

145. Oster SK, Marhin WW, Asker C, Facchini LM, Dion PA, Funa K, Post M, Sedivy JM, Penn LZ: **Myc is an essential negative regulator of platelet-derived growth factor beta receptor expression.** *Mol Cell Biol* 2000, **20:**6768-6778.

146. Penn LJ, Brooks MW, Laufer EM, Land H: **Negative autoregulation of c-myc transcription.** *Embo J* 1990, **9:**1113-1121.

147. Facchini LM, Chen S, Marhin WW, Lear JN, Penn LZ: **The Myc negative autoregulation mechanism requires Myc-Max association and involves the c-myc P2 minimal promoter.** *Mol Cell Biol* 1997, **17:**100-114.

148. Chiang YC, Teng SC, Su YN, Hsieh FJ, Wu KJ: **c-Myc directly regulates the transcription of the NBS1 gene involved in DNA double-strand break repair.** *J Biol Chem* 2003, **278:**19286-19291.

149. Greasley PJ, Bonnard C, Amati B: **Myc induces the nucleolin and BN51 genes: possible implications in ribosome biogenesis.** *Nucleic Acids Res* 2000, **28:**446-453.

150. Shimono A, Okuda T, Kondoh H: **N-myc-dependent repression of ndr1, a gene identified by direct subtraction of whole mouse embryo cDNAs between wild type and N-myc mutant.** *Mech Dev* 1999, **83:**39-52.

151. Tsuneoka M, Teye K, Arima N, Soejima M, Otera H, Ohashi K, Koga Y, Fujita H, Shirouzu K, Kimura H, Koda Y: **A novel Myc-target gene, mimitin, that is involved in cell proliferation of esophageal squamous cell carcinoma.** *J Biol Chem* 2005, **280:**19977-19985.

152. Godfried MB, Veenstra M, v Sluis P, Boon K, v Asperen R, Hermus MC, v Schaik BD, Voute TP, Schwab M, Versteeg R, Caron HN: **The N-myc and c-myc downstream pathways include the chromosome 17q genes nm23-H1 and nm23-H2.** *Oncogene* 2002, **21:**2097-2101.

153. Pal S, Yun R, Datta A, Lacomis L, Erdjument-Bromage H, Kumar J, Tempst P, Sif S: **mSin3A/histone deacetylase 2- and PRMT5-containing Brg1 complex is involved in transcriptional repression of the Myc target gene cad.** *Mol Cell Biol* 2003, **23:**7475-7487.

154. Biroccio A, Benassi B, Filomeni G, Amodei S, Marchini S, Chiorino G, Rotilio G, Zupi G, Ciriolo MR: **Glutathione influences c-Myc-induced apoptosis in M14 human melanoma cells.** *J Biol Chem* 2002, **277:**43763-43770.

155. Eberhardy SR, D'Cunha CA, Farnham PJ: **Direct examination of histone acetylation on Myc target genes using chromatin immunoprecipitation.** *J Biol Chem* 2000, **275:**33798-33805.

156. Bello-Fernandez C, Packham G, Cleveland JL: **The ornithine decarboxylase gene is a transcriptional target of c-Myc.** *Proc Natl Acad Sci U S A* 1993, **90:**7804-7808.

157. Wagner AJ, Meyers C, Laimins LA, Hay N: **c-Myc induces the expression and activity of ornithine decarboxylase.** *Cell Growth Differ* 1993, **4:**879-883.

158. Law GL, Itoh H, Law DJ, Mize GJ, Merchant JL, Morris DR: **Transcription factor ZBP-89 regulates the activity of the ornithine decarboxylase promoter.** *J Biol Chem* 1998, **273:**19955-19964.

159. Wanzel M, Kleine-Kohlbrecher D, Herold S, Hock A, Berns K, Park J, Hemmings B, Eilers M: **Akt and 14-3-3eta regulate Miz1 to control cell-cycle arrest after DNA damage.** *Nat Cell Biol* 2005, **7:**30-41.

160. Riu E, Ferre T, Mas A, Hidalgo A, Franckhauser S, Bosch F: **Overexpression of c-myc in diabetic mice restores altered expression of the transcription factor genes that regulate liver metabolism.** *Biochem J* 2002, **368:**931-937.

161. Moll J, Schmid P, Sansig G, van der Putten H: **The pattern of prothymosin alpha gene expression coincides with that of myc proto-oncogenes during mouse embryogenesis.** *Histochem J* 1996, **28:**45-52.

162. Eilers M, Schirm S, Bishop JM: **The MYC protein activates transcription of the alpha-prothymosin gene.** *Embo J* 1991, **10:**133-141.

163. Walhout AJ, van der Vliet PC, Timmers HT: **Sequences flanking the E-box contribute to cooperative binding by c-Myc/Max heterodimers to adjacent binding sites.** *Biochim Biophys Acta* 1998, **1397:**189-201.

164. Gaubatz S, Meichle A, Eilers M: **An E-box element localized in the first intron mediates regulation of the prothymosin alpha gene by c-myc.** *Mol Cell Biol* 1994, **14:**3853-3862.

165. Tsuneoka M, Nakano F, Ohgusu H, Mekada E: **c-myc activates RCC1 gene expression through E-box elements.** *Oncogene* 1997, **14:**2301-2311.

166. Zou H, Lifshitz LM, Tuft RA, Fogarty KE, Singer JJ: **Visualization of Ca2+ entry through single stretch-activated cation channels.** *Proc Natl Acad Sci U S A* 2002, **99:**6404-6409.

167. Nikiforov MA, Chandriani S, O'Connell B, Petrenko O, Kotenko I, Beavis A, Sedivy JM, Cole MD: **A functional screen for Myc-responsive genes reveals serine hydroxymethyltransferase, a major source of the one-carbon unit for cell metabolism.** *Mol Cell Biol* 2002, **22:**5793-5800.

168. Zuo F, Kaminski N, Eugui E, Allard J, Yakhini Z, Ben-Dor A, Lollini L, Morris D, Kim Y, DeLustro B, et al: **Gene expression analysis reveals matrilysin as a key regulator of pulmonary fibrosis in mice and humans.** *Proc Natl Acad Sci U S A* 2002, **99:**6292-6297.

169. O'Donnell KA, Yu D, Zeller KI, Kim JW, Racke F, Thomas-Tikhonenko A, Dang CV: **Activation of transferrin receptor 1 by c-Myc enhances cellular proliferation and tumorigenesis.** *Mol Cell Biol* 2006, **26:**2373-2386.

170. Pusch O, Soucek T, Hengstschlager-Ottnad E, Bernaschek G, Hengstschlager M: **Cellular targets for activation by c-Myc include the DNA metabolism enzyme thymidine kinase.** *DNA Cell Biol* 1997, **16:**737-747.

171. Kim JW, Zeller KI, Wang Y, Jegga AG, Aronow BJ, O'Donnell KA, Dang CV: **Evaluation of myc E-box phylogenetic footprints in glycolytic genes by chromatin immunoprecipitation assays.** *Mol Cell Biol* 2004, **24:**5923-5936.

172. Jones DT, Trowbridge IS, Harris AL: **Effects of transferrin receptor blockade on cancer cell proliferation and hypoxia-inducible factor function and their differential regulation by ascorbate.** *Cancer Res* 2006, **66:**2749-2756.

173. Wu Q, Kirschmeier P, Hockenberry T, Yang TY, Brassard DL, Wang L, McClanahan T, Black S, Rizzi G, Musco ML, et al: **Transcriptional regulation during p21WAF1/CIP1-induced apoptosis in human ovarian cancer cells.** *J Biol Chem* 2002, **277:**36329-36337.

174. Chang BD, Watanabe K, Broude EV, Fang J, Poole JC, Kalinichenko TV, Roninson IB: **Effects of p21Waf1/Cip1/Sdi1 on cellular gene expression: implications for carcinogenesis, senescence, and age-related diseases.** *Proc Natl Acad Sci U S A* 2000, **97:**4291-4296.

175. Wu RC, Schonthal AH: **Activation of p53-p21waf1 pathway in response to disruption of cell-matrix interactions.** *J Biol Chem* 1997, **272:**29091-29098.

176. Zhang JS, Wang L, Huang H, Nelson M, Smith DI: **Keratin 23 (K23), a novel acidic keratin, is highly induced by histone deacetylase inhibitors during differentiation of pancreatic cancer cells.** *Genes Chromosomes Cancer* 2001, **30:**123-135.

177. Arima Y, Hirota T, Bronner C, Mousli M, Fujiwara T, Niwa S, Ishikawa H, Saya H: **Down-regulation of nuclear protein ICBP90 by p53/p21Cip1/WAF1-dependent DNA-damage checkpoint signals contributes to cell cycle arrest at G1/S transition.** *Genes Cells* 2004, **9:**131-142.

178. Wang A, Schneider-Broussard R, Kumar AP, MacLeod MC, Johnson DG: **Regulation of BRCA1 expression by the Rb-E2F pathway.** *J Biol Chem* 2000, **275:**4532-4536.

179. Markey MP, Angus SP, Strobeck MW, Williams SL, Gunawardena RW, Aronow BJ, Knudsen ES: **Unbiased analysis of RB-mediated transcriptional repression identifies novel targets and distinctions from E2F action.** *Cancer Res* 2002, **62:**6587-6597.

180. Ren B, Cam H, Takahashi Y, Volkert T, Terragni J, Young RA, Dynlacht BD: **E2F integrates cell cycle progression with DNA repair, replication, and G(2)/M checkpoints.** *Genes Dev* 2002, **16:**245-256.

181. Kumar S, Cakouros D: **Transcriptional control of the core cell-death machinery.** *Trends Biochem Sci* 2004, **29:**193-199.

182. Stanelle J, Putzer BM: **E2F1-induced apoptosis: turning killers into therapeutics.** *Trends Mol Med* 2006, **12:**177-185.

183. Kohn KW: **Molecular interaction map of the mammalian cell cycle control and DNA repair systems.** *Mol Biol Cell* 1999, **10:**2703-2734.

184. DeGregori J: **The genetics of the E2F family of transcription factors: shared functions and unique roles.** *Biochim Biophys Acta* 2002, **1602:**131-150.

185. Ma Y, Yuan J, Huang M, Jove R, Cress WD: **Regulation of the cyclin D3 promoter by E2F1.** *J Biol Chem* 2003, **278:**16770-16776.

186. Hartman J, Muller P, Foster JS, Wimalasena J, Gustafsson JA, Strom A: **HES-1 inhibits 17beta-estradiol and heregulin-beta1-mediated upregulation of E2F-1.** *Oncogene* 2004, **23:**8826-8833.

187. Konishi Y, Bonni A: **The E2F-Cdc2 cell-cycle pathway specifically mediates activity deprivation-induced apoptosis of postmitotic neurons.** *J Neurosci* 2003, **23:**1649-1658.

188. Arata Y, Fujita M, Ohtani K, Kijima S, Kato JY: **Cdk2-dependent and -independent pathways in E2F-mediated S phase induction.** *J Biol Chem* 2000, **275:**6337-6345.

189. Gartel AL, Najmabadi F, Goufman E, Tyner AL: **A role for E2F1 in Ras activation of p21(WAF1/CIP1) transcription.** *Oncogene* 2000, **19:**961-964.

190. Zaika A, Irwin M, Sansome C, Moll UM: **Oncogenes induce and activate endogenous p73 protein.** *J Biol Chem* 2001, **276:**11310-11316.

191. Hiyama H, Iavarone A, LaBaer J, Reeves SA: **Regulated ectopic expression of cyclin D1 induces transcriptional activation of the cdk inhibitor p21 gene without altering cell cycle progression.** *Oncogene* 1997, **14:**2533-2542.

192. Gartel AL, Goufman E, Tevosian SG, Shih H, Yee AS, Tyner AL: **Activation and repression of p21(WAF1/CIP1) transcription by RB binding proteins.** *Oncogene* 1998, **17:**3463-3469.

193. Blais A, Monte D, Pouliot F, Labrie C: **Regulation of the human cyclin-dependent kinase inhibitor p18INK4c by the transcription factors E2F1 and Sp1.** *J Biol Chem* 2002, **277:**31679-31693.

194. Carrassa L, Broggini M, Vikhanskaya F, Damia G: **Characterization of the 5'flanking region of the human Chk1 gene: identification of E2F1 functional sites.** *Cell Cycle* 2003, **2:**604-609.

195. Luciakova K, Barath P, Li R, Zaid A, Nelson BD: **Activity of the human cytochrome c1 promoter is modulated by E2F.** *Biochem J* 2000, **351:**251-256.

196. Li Y, Slansky JE, Myers DJ, Drinkwater NR, Kaelin WG, Farnham PJ: **Cloning, chromosomal location, and characterization of mouse E2F1.** *Mol Cell Biol* 1994, **14:**1861-1869.

197. Christensen J, Cloos P, Toftegaard U, Klinkenberg D, Bracken AP, Trinh E, Heeran M, Di Stefano L, Helin K: **Characterization of E2F8, a novel E2F-like cell-cycle regulated repressor of E2F-activated transcription.** *Nucleic Acids Res* 2005, **33:**5458-5470.

198. Croxton R, Ma Y, Song L, Haura EB, Cress WD: **Direct repression of the Mcl-1 promoter by E2F1.** *Oncogene* 2002, **21:**1359-1369.

199. Yoshida K, Inoue I: **Expression of MCM10 and TopBP1 is regulated by cell proliferation and UV irradiation via the E2F transcription factor.** *Oncogene* 2004, **23:**6250-6260.

200. Joshi B, Ordonez-Ercan D, Dasgupta P, Chellappan S: **Induction of human metallothionein 1G promoter by VEGF and heavy metals: differential involvement of E2F and metal transcription factors.** *Oncogene* 2005, **24:**2204-2217.

201. Hershko T, Ginsberg D: **Up-regulation of Bcl-2 homology 3 (BH3)-only proteins by E2F1 mediates apoptosis.** *J Biol Chem* 2004, **279:**8627-8634.

202. Kalma Y, Marash L, Lamed Y, Ginsberg D: **Expression analysis using DNA microarrays demonstrates that E2F-1 up-regulates expression of DNA replication genes including replication protein A2.** *Oncogene* 2001, **20:**1379-1387.

203. Iwanaga R, Komori H, Ishida S, Okamura N, Nakayama K, Nakayama KI, Ohtani K: **Identification of novel E2F1 target genes regulated in cell cycle-dependent and independent manners.** *Oncogene* 2006, **25:**1786-1798.

204. Mousli M, Hopfner R, Abbady AQ, Monte D, Jeanblanc M, Oudet P, Louis B, Bronner C: **ICBP90 belongs to a new family of proteins with an expression that is deregulated in cancer cells.** *Br J Cancer* 2003, **89:**120-127.

205. Unoki M, Nishidate T, Nakamura Y: **ICBP90, an E2F-1 target, recruits HDAC1 and binds to methyl-CpG through its SRA domain.** *Oncogene* 2004, **23:**7601-7610.

206. Vernell R, Helin K, Muller H: **Identification of target genes of the p16INK4A-pRB-E2F pathway.** *J Biol Chem* 2003, **278:**46124-46137.

207. Hilakivi-Clarke L: **Estrogens, BRCA1, and breast cancer.** *Cancer Res* 2000, **60:**4993-5001.

208. Zhang HS, Dean DC: **Rb-mediated chromatin structure regulation and transcriptional repression.** *Oncogene* 2001, **20:**3134-3138.

209. Marshall TW, Link KA, Petre-Draviam CE, Knudsen KE: **Differential requirement of SWI/SNF for androgen receptor activity.** *J Biol Chem* 2003, **278:**30605-30613.

210. Dasgupta P, Betts V, Rastogi S, Joshi B, Morris M, Brennan B, Ordonez-Ercan D, Chellappan S: **Direct binding of apoptosis signal-regulating kinase 1 to retinoblastoma protein: novel links between apoptotic signaling and cell cycle machinery.** *J Biol Chem* 2004, **279:**38762-38769.

211. Lemercier C, Duncliffe K, Boibessot I, Zhang H, Verdel A, Angelov D, Khochbin S: **Involvement of retinoblastoma protein and HBP1 in histone H1(0) gene expression.** *Mol Cell Biol* 2000, **20:**6627-6637.

212. Di Matteo G, Fuschi P, Zerfass K, Moretti S, Ricordy R, Cenciarelli C, Tripodi M, Jansen-Durr P, Lavia P: **Transcriptional control of the Htf9-A/RanBP-1 gene during the cell cycle.** *Cell Growth Differ* 1995, **6:**1213-1224.

213. Park K, Choe J, Osifchin NE, Templeton DJ, Robbins PD, Kim SJ: **The human retinoblastoma susceptibility gene promoter is positively autoregulated by its own product.** *J Biol Chem* 1994, **269:**6083-6088.
